# Data-driven modelling of IRCU patient flow during the COVID-19 pandemic

**DOI:** 10.1016/j.csbj.2025.10.017

**Published:** 2025-10-17

**Authors:** Ana Carmen Navas-Ortega, José Antonio Sánchez-Martínez, Paula García-Flores, Concepción Morales-García, Rene Fabregas

**Affiliations:** a Department of Pneumology, University Hospital Virgen de Las Nieves, Granada, Spain; b Biosanitary Research Institute of Granada-Ibs, Granada, Spain; c Department of Applied Mathematics and Modeling Nature (MNat) Research Unit, Faculty of Sciences, University of Granada, Granada, Spain

**Keywords:** Intermediate respiratory care unit, COVID-19, Non-invasive ventilation, Respiratory failure, Clinical outcomes, Patient flow dynamics, Mathematical modelling, Clinical staff, Healthcare operations

## Abstract

**Background:**

Intermediate Respiratory Care Units (IRCUs) function as vital intermediaries between general wards and Intensive Care Units (ICUs), particularly during crises such as the COVID-19 pandemic. A unit’s effectiveness depends on its structure, protocols, and clinical expertise. In this study, we assessed the clinical outcomes and operational dynamics of a new IRCU that implemented a specialist staffing model during the pandemic in Spain.

**Methods:**

We conducted a prospective cohort study at the UHVN IRCU (Granada, Spain) from April to August 2021, enrolling 249 adult patients with COVID-19-associated respiratory failure. We collected data on patient demographics, Non-Invasive Ventilation (NIV) use, length of stay (LOS), and outcomes, including ICU transfer, mortality, and recovery. We then analysed these outcomes stratified by NIV status. Furthermore, we developed and calibrated a compartmental Ordinary Differential Equation (ODE) model and an empirical LOS-based convolution model to simulate patient flow dynamics under scenarios of admission surges and varying care efficiency.

**Results:**

The cohort’s median age was 51 years, and 31 % (n=77) required NIV. Patients requiring NIV were significantly older than those who did not (median 61 vs 42 years, p<0.001). Overall, 8 % of patients (n=20) were subsequently transferred to the ICU, and 3 % (n=7) died within the IRCU. Notably, no patients managed without NIV required ICU transfer or died. Among the 77 high-risk patients who received NIV, 68 % recovered within the IRCU without needing ICU escalation. Our ODE modelling accurately reproduced aggregate outcomes and demonstrated that simulated admission surges placed the system under significant strain, which enhanced recovery efficiency partially mitigated. The LOS-based modelling yielded consistent peak occupancy estimates.

**Conclusion:**

This IRCU, characterised by specialist clinical staffing, demonstrated effective management of severe COVID-19 respiratory failure. We observed high recovery rates, particularly among NIV patients, which eased pressure on ICU resources. Our dynamic modelling confirmed the unit’s vulnerability to admission surges but also quantified the positive impact of efficient care. These findings underscore the importance of well-structured and expertly staffed IRCUs in pandemic response and the broader provision of respiratory care.

## Graphical Abstract

**Figure:**
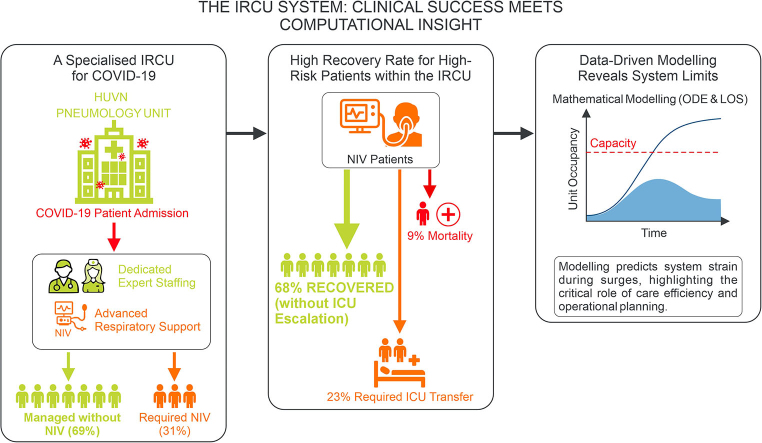

## Nomenclature

**Abbreviations**
BiPAP: Bilevel Positive Airway Pressure
CEIM/CEI: Comité de Ética de Investigación
CI: Confidence Interval
CPAP: Continuous Positive Airway Pressure
FiO2: Fractional Inspired Oxygen
GP: Gaussian Process
HFNO: High-Flow Nasal Oxygen
ICU: Intensive Care Unit
IQR: Interquartile Range
IRCU: Intermediate Respiratory Care Unit
LOS: Length of Stay
NIV: Non-Invasive Ventilation
ODE: Ordinary Differential Equation
OR: Odds Ratio
PMF: Probability Mass Function
RR: Risk Ratio
SEPAR: Sociedad Española de Neumología
UHVN: University Hospital Virgen de las Nieves

## 1 Introduction

The management of severe respiratory failure is a fundamental aspect of critical care medicine [1], a challenge that the pressures of the COVID-19 pandemic highlighted [2], [3], [4]. In tiered hospital care, Intermediate Respiratory Care Units (IRCUs) are a critical component [5], [6], bridging the gap between the Intensive Care Unit (ICU) and the general ward [7], [8], [9], [10], [11], [12], [13], [14]. Clinicians originally developed IRCUs to provide non-invasive respiratory support (NIV) [5], [6], [15], [16], [17] such as BiPAP/CPAP and high-flow nasal oxygen (HFNO) [1], [18], [19], IRCUs were indispensable during the pandemic [20], [21]. They function both upstream to prevent ICU admissions and downstream to facilitate ICU discharge [22], [23], [24], [25]. Their benefits, including reduced need for invasive mechanical ventilation [26], [27], [28] and associated healthcare costs [17], [29], are well-recognised [7], [30], [31]

Despite the proliferation of IRCUs and evidence of their utility [14], [32], [33], the determinants of their success remain poorly defined [12], [34]. While the literature frequently discusses structural factors and patient selection [7], [8], [35], the impact of specialist clinical staffing models—utilising physicians and nurses trained in advanced respiratory care [36], [37], [38]—has received less systematic attention. Although a large body of evidence shows that general nurse staffing levels and specialist physician involvement improve patient outcomes [39], [40], [41], [42], [43], researchers have not systematically evaluated the specific contribution of targeted multidisciplinary expertise in an IRCU environment [8], [18], [44], [45], [46], [47], a gap highlighted in recent reviews [14], [36], [48]. This represents a significant gap in understanding optimal intermediate care delivery.

**Fig. 6:**
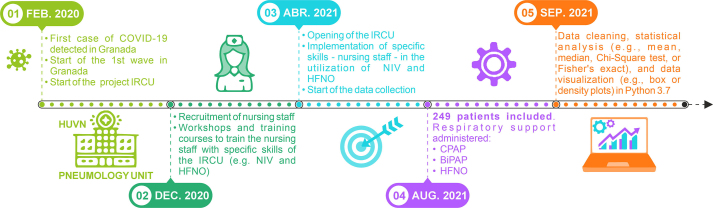
Timeline of UHVN IRCU Implementation and Study Execution. Key milestones include: (01) Feb 2020: Project initiation driven by COVID-19 emergence. (02) Dec 2020: Clinical staff preparation, including nursing staff recruitment and specialised training (NIV/HFNO). (03) Apr 2021: Official IRCU opening and commencement of data collection. (04) Aug 2021: End of the patient inclusion period, encompassing 249 patients who received respiratory support (CPAP, BiPAP, HFNO). (05) Sep 2021: Initiation of data cleaning and statistical analysis.

**Fig. 7:**
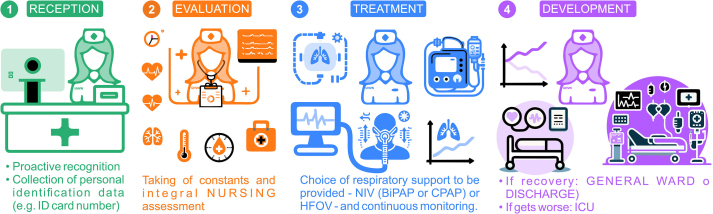
Patient Management Workflow and Key Clinical Team Actions in the UHVN IRCU. The process encompasses four main stages: (1) **Reception**: Initial patient identification and proactive recognition. (2) **Evaluation**: Collection of vital signs and integral clinical assessment (including nursing). (3) **Treatment**: Selection and administration of respiratory support (NIV: CPAP, BiPAP, HFNO) with continuous multidisciplinary monitoring. (4) **Development**: Monitoring patient trajectory towards outcomes (Recovery: transfer to General Ward or Discharge; Worsening: transfer to ICU; or Exitus).

We hypothesise that IRCUs staffed by respiratory specialists achieve superior clinical outcomes and patient flow optimisation during pandemic conditions [18], [22], [23], [26], [38], [49], [50], [51], [52]. We therefore analysed the clinical effectiveness and operational dynamics of a new IRCU at the University Hospital Virgen de las Nieves (UHVN), Granada, during its first five months of operation (April to August 2021). The unit featured 24/7 respiratory physician coverage and nurses with specific training (Fig. 6, Fig. 7). We quantified patient outcomes, including length of stay and ICU transfer rates [24], [29], and elucidated its performance characteristics [11], [29] to provide data-driven insights into this specialist staffing model [36], [43], [53], [54].

This work’s principal contribution is a methodological advance over prior descriptive accounts [15], [22], [55], combining detailed statistical analysis (Section 2.1) with a novel quantitative framework based on dynamic systems modelling [56], [57], [58]. This dual approach elucidates the temporal dynamics of patient cohorts within the IRCU [59], [60], enabling system simulation under variable conditions [61], [62] and the identification of critical parameters governing operational efficiency [63], [64], [65]. By linking a specialist-staffed care model to clinical and dynamic results, this study provides a systematic evaluation that addresses the existing knowledge gap regarding the impact of such models in intermediate respiratory care [36], [38], [66].

First, we detail the clinical characteristics and outcomes of the 249-patient cohort (Section 2.1). Secondly, we introduce a compartmental Ordinary Differential Equation (ODE) model to simulate patient flow between NIV and non-NIV states, leading to outcomes (mortality, recovery, or ICU transfer), under various operational scenarios (Section 2.2). Third, we present a complementary occupancy model based on the empirical Length-of-Stay distribution (Section 2.3). Finally, we discuss the implications of our findings for clinical practice and healthcare policy, contextualising our results within intermediate care medicine (Sections 3 and 4).

## 2 Results

### 2.1 Cohort characteristics and clinical outcomes

**Fig. 1:**
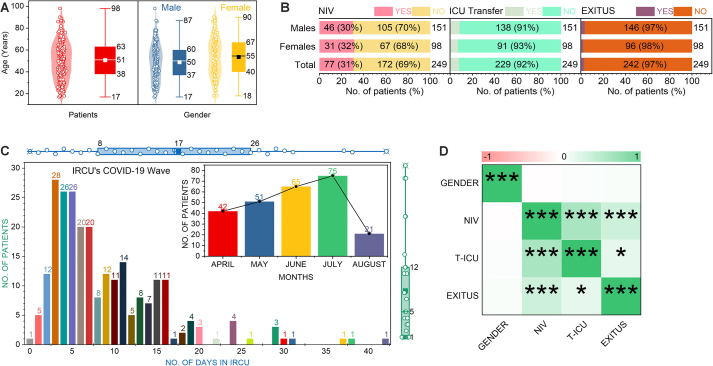
Patient characteristics and clinical outcomes in the IRCU cohort (n=249). (**A**) Violin plots show the distribution, with inner boxes indicating the median and IQR. Age distribution for the overall cohort (median 51, IQR: 38–63) and stratified by gender (male: median 50, IQR: 37–60; female: median 55, IQR: 40–67). (**B**) Distribution of key outcomes for the total cohort, stratified by gender. Overall, 31 % of patients (n=77) required NIV, 8 % (n=20) were transferred to the ICU, and 3 % (n=7) died. A minor data inconsistency exists between gender-stratified and total counts for NIV use. (**C**) Distribution of length of stay (LOS) in the IRCU (median 5 days, IQR: 3–8). The inset shows monthly admissions from April to August 2021. (**D**) Correlation matrix for binary clinical variables.Significance levels: ns (p≥0.05); *p<0.05; **p<0.01; ***p<0.001.

The analysis included 249 patients admitted to the IRCU between April and August 2021. The cohort’s median age was 51 years (Interquartile Range [IQR]: 38–63), with a range of 17 to 98 years (Fig. 1A, left panel). A significant gender difference was observed in age distribution (Mann-Whitney U = 3405, p = 0.023); female patients (n=98) had a higher median age (55 years, IQR: 40–67) than male patients (n=151; median 50, IQR: 37–60) (Fig. 1A, right panel).

**Fig. 2:**
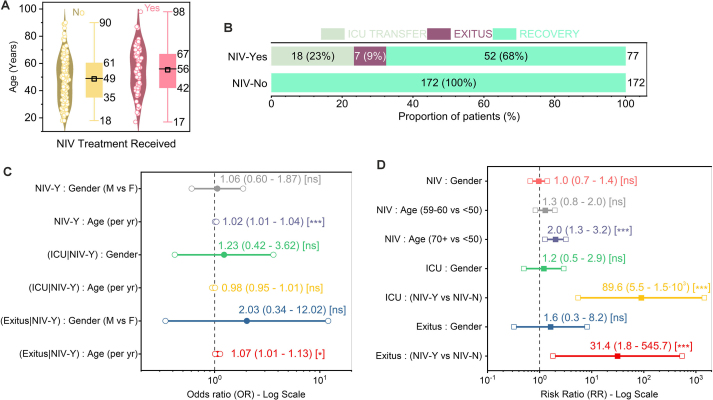
Analysis of patient outcomes and multivariable associations, stratified by NIV treatment status. (**A**) Age distribution stratified by NIV treatment status, showing patients requiring NIV (n=77) were significantly older (median 61, IQR 56–67) than those who did not (n=172; median 42, IQR 35–49). (**B**) Distribution of final, mutually exclusive outcomes. Of 77 patients requiring NIV, 23 % (n=18) were transferred to the ICU, 9 % (n=7) died, and 68 % (n=52) recovered. All 172 patients managed without NIV recovered. Outcomes are defined in order of precedence: ICU transfer, then mortality without prior ICU transfer, then recovery. (**C**) Adjusted Odds Ratios (OR) from logistic regression, adjusted for age and gender. The reference group for gender is female. (**D**) Crude Risk Ratios (RR) comparing risks between groups. Reference groups are: female (for gender), age <50 years, and non-NIV. The high RRs for NIV reflect significant confounding by indication.Significance levels: ns (p≥0.05); *p<0.05; **p<0.01; ***p<0.001.

Overall, 77 patients (31 %) required NIV. Subsequently, 8 % of patients (n=20) were transferred to the ICU and 3 % (n=7) died within the IRCU (Fig. 1B, Total rows). Although absolute counts suggested similar patterns, formal statistical tests confirmed no significant association between gender and NIV use, ICU transfer, or mortality (Fig. 2C and D; all *p* > 0.05, not significant [ns]).

The median length of stay (LOS) in the IRCU was 5 days (IQR: 1–12), but showed considerable variability, with some stays exceeding 40 days (Fig. 1C). Monthly admissions peaked in June 2021 (n=75), likely reflecting regional pandemic activity (Fig. 1C inset). Correlation analysis confirmed significant positive associations (*p*<0.001) between ICU transfer (T-ICU) and mortality, as well as between NIV use and both these adverse outcomes, suggesting greater severity in these patients (Fig. 1D). Gender was not significantly correlated with these variables (p>0.05).

Stratification by NIV status revealed significant clinical differences. Patients requiring NIV (n=77) were significantly older than those managed without it (n=172): median age 61 (IQR: 56–67) vs 42 (IQR: 35–49) years, respectively (Fig. 2A). Age was a strong predictor of NIV requirement; adjusted logistic regression showed increased odds of receiving NIV per year of age (OR = 1.02, 95 % CI: 1.01–1.04, *p* < 0.001), and patients aged ≥70 years had double the risk compared to those <50 (RR = 2.0, 95 % CI: 1.3–3.2, *p* < 0.001) (Fig. 2C, D). Gender was not significantly associated with NIV use (Fig. 2C, D).

Outcomes differed starkly between the groups (Fig. 2B). All 172 patients managed without NIV recovered without ICU transfer or in-unit mortality. In contrast, of the 77 higher-risk patients requiring NIV, 23 % (n=18) were transferred to the ICU, 9 % (n=7) died without prior ICU transfer, and 68 % (n=52) recovered. The resulting high crude Risk Ratios for ICU transfer (RR = 89.6, 95 % CI: 5.5–1464, *p* < 0.001) and mortality (RR = 31.4, 95 % CI: 1.8–546, *p* < 0.001) in NIV users must be interpreted in light of significant confounding by indication (Fig. 2D). Adjusted analyses confirmed that older age remained an important risk factor for mortality within the NIV group (OR = 1.07 per year, 95 % CI: 1.01–1.13, *p*=0.013), while gender showed no significant association with outcomes in this subgroup (*p* > 0.05) (Fig. 2C, D). Crucially, the recovery of 68 % of NIV-treated patients without ICU escalation underscores the IRCU’s effectiveness in managing this high-risk population.

### 2.2 Data-driven modeling

While the preceding statistical analysis identified key prognostic factors (Section 2.1), such static methods offer limited insight into the temporal dynamics of patient flow. To gain a mechanistic understanding of the interplay between admissions, care pathways, and operational efficiency, we therefore developed a compartmental model based on Ordinary Differential Equations (ODEs), a standard technique in epidemiology and healthcare operations [63].

The model tracks the number of patients in distinct states over time t: patients in the IRCU not requiring Non-Invasive Ventilation, X(t); those currently receiving NIV, Y(t); and the cumulative number of patients transferred to the ICU, Z(t); experiencing in-IRCU mortality, W(t); or recovering, R(t).

The temporal evolution of these states is governed by the following system of ODEs:

(1) $$\frac{dX}{dt}=A(t)-(\alpha +\rho +\eta +\nu )X(t),$$

(2) $$\frac{dY}{dt}=\alpha X(t)-[\gamma (1-\gamma_{0})+\theta (t)+\varepsilon (1-\varepsilon_{0})]Y(t),$$

(3) $$\frac{dZ}{dt}=\gamma (1-\gamma_{0})Y(t)+\eta X(t),$$

(4) $$\frac{dW}{dt}=\varepsilon (1-\varepsilon_{0})Y(t)+\nu X(t),$$

(5) $$\frac{dR}{dt}=\theta (t)Y(t)+\rho X(t).$$

Here, A(t) is the time-dependent admission rate. For non-NIV patients (X), transition rates are: α (NIV initiation), ρ (recovery), η (ICU transfer), and ν (mortality). For NIV patients (Y), rates are: γ (ICU transfer), θ(t) (time-dependent recovery), and ε (mortality). The cumulative states Z(t), W(t), and R(t) track outcomes. The model considers only inflow to the ICU; subsequent ICU dynamics are outside its scope. A complete derivation, parameter definitions, and estimation procedures are provided in the Supplementary Material.

The model’s baseline transition rates were calibrated using observed outcome proportions from the UHVN cohort (n=249). Based on the finding that patients managed without NIV experienced no adverse outcomes (Fig. 2B), the direct transition rates to ICU and mortality from the non-NIV state were structurally constrained to zero: η=0 and ν=0. For the remaining pathways, outcome proportions were converted into rates [days^-1^] by incorporating assumed average lengths of stay (LOS) for the non-NIV ($\tau_{X}=7$ days) and NIV ($\tau_{Y}=10$ days) states. This calibration aligns the model’s parameters with aggregate cohort behaviour, a prerequisite for credible simulation [67]. The detailed methodology is documented in the Supplementary Material.

#### 2.2.1 Simulation of IRCU patient flow dynamics

**Fig. 3:**
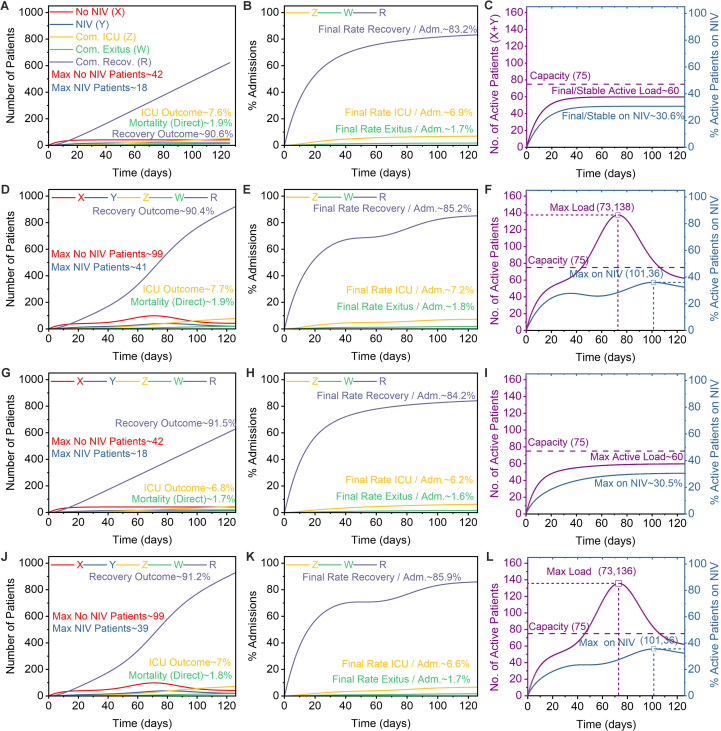
Dynamic outputs of the ODE model under four scenarios. The figure compares the 126-day system dynamics across four scenarios, tracking active patients (X: No NIV; Y: NIV) and cumulative outcomes (Z: ICU; W: mortality; R: recovery). Each scenario (row) is consistently visualised across three columns: (1) absolute patient trajectories and outcome proportions (A, D, G, J); (2) cumulative outcome rates normalised by admissions (B, E, H, K); and (3) total active patient load (X+Y) relative to a 75-bed capacity threshold (C, F, I, L). The simulations utilise calibrated parameters from cohort data (Suppl. Section S5), with η and ν set to 0. The specific scenarios are: (A–C) Baseline (A1): constant admission ($A(t)=A_{avg}$) and recovery ($\theta (t)=\theta_{0}$) rates. (D–F) Variable Admissions (B1): a Gaussian admission surge (A(t)) superimposed on the baseline, with constant recovery. (G–I) Variable Recovery (B2): a transient improvement in recovery efficiency ($\theta (t)=\theta_{0}+0.15e^{-0.05\;t}$), with constant admissions. (J-L) Combined (B3): a surge occurring concurrently with efficiency gains (variable A(t) and θ(t) as in B1 and B2).

To validate the calibrated ODE model’s ability to replicate empirical observations, we executed a baseline autonomous simulation (A1) representing average operational conditions. This scenario used a constant admission rate ($A_{avg}$) and the empirically derived parameters (Supplementary Material, Section S5), including the structural constraint of no direct mortality or ICU transfer from the non-NIV state (η=0,ν=0). The simulation successfully reproduced the system’s aggregate behaviour, evolving to a steady state (Fig. 3A–C) where the active census stabilised at approximately 60 patients (non-NIV X ≈ 42, NIV Y ≈ 18)—well below an illustrative 75-bed capacity threshold—with 30.6 % requiring NIV. With recovery being the predominant pathway, the final outcome proportions at day 126—7.6 % ICU transfer, 1.9 % mortality, and 90.6 % recovery—demonstrated good concordance with the observed clinical data (Fig. 3A vs. B). This concordance confirms the model’s baseline validity, with cumulative outcome rates per admission stabilising at values consistent with the cohort: 6.9 % for ICU transfer, 1.7 % for mortality, and 83.2 % for recovery (Fig. 3B).

Having validated the baseline model, we then used it to isolate the impact of admission volatility by simulating a surge scenario (A(t), Simulation B1) while keeping operational efficiency constant ($\theta (t)=\theta_{0}$) (Fig. 3D–F). The simulation revealed two distinct effects. First, the surge created severe transient strain on resources: the peak NIV census more than doubled (from 18 to 41 patients), and the total active patient load peaked at 138, substantially exceeding the 75-bed capacity. Second, despite this acute capacity crisis, the surge did not fundamentally alter the long-term outcome distribution; the final proportions of ICU transfer (7.7 %), mortality (1.9 %), and recovery (90.4 %) remained nearly identical to the baseline. This crucial finding demonstrates that, under constant efficiency, admission surges manifest as a transient strain on capacity rather than a degradation of overall patient outcome pathways.

Building on the observed 68 % NIV success rate (Fig. 2B), we designed a simulation (B2) to quantify the specific impact of enhanced clinical efficiency (Fig. 3G–I). In this scenario, we introduced a time-varying improvement to the NIV recovery rate ($\theta (t)=\theta_{0}+\Delta \theta e^{-\lambda t}$) while holding the admission rate constant. The model predicted a clear, albeit modest, improvement in patient outcomes compared to the baseline. Specifically, final ICU transfers fell from 7.6 % to 6.8 % and mortality from 1.9 % to 1.7 %, while recoveries improved from 90.6 % to 91.5 % (Fig. 3G). This favourable shift was also evident in the outcome rates per admission (Fig. 3H). Crucially, because the admission rate was constant, the active patient load dynamics remained unchanged from the baseline, stabilising at approximately 60 patients—below the illustrative capacity—with around 30.5 % requiring NIV. This result isolates the effect of care efficiency, demonstrating that it can improve patient trajectories even without a reduction in the overall system’s census (Fig. 3I).

Our final simulation combined an admission surge with improved care efficiency (B3, Fig. 3J–L), revealing a crucial distinction between managing patient outcomes and managing patient volume. The results showed that while enhanced recovery efficiency (θ(t)) modestly improved final outcome proportions compared to a surge with constant efficiency (B1)—reducing ICU transfers (7.0 % vs. 7.7 %) and mortality (1.8 % vs. 1.9 %) while increasing recoveries (91.2 % vs. 90.4 %)—it did not prevent the acute resource strain. The active patient load still peaked at approximately 136 (max Y ≈ 39), a level that substantially surpassed the unit’s capacity and closely mirrored the surge-only scenario. This finding demonstrates that while improvements in care efficiency are valuable for patient trajectories, they are insufficient to alleviate the peak operational burden imposed by a major admission surge.

**Fig. 4:**
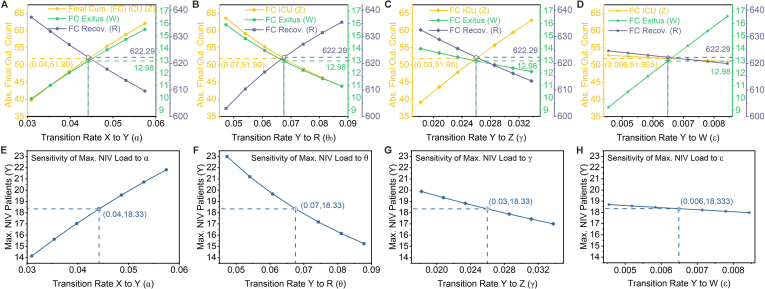
Sensitivity analysis of the autonomous model’s key parameters.The figure illustrates the impact of varying the four main transition rates—α, $\theta_{0}$, γ, and ε—by ±30 % around their baseline values. The analysis was performed on the autonomous model (constant admission rate and recovery). Markers and dashed lines indicate the baseline reference point, defined by parameter values of α≈0.044, $\theta_{0}\approx 0.068$, γ≈0.023, and ε≈0.009 days^-1^, which corresponds to model outputs of approximately 13 ICU transfers (Z), 52 mortalities (W), 622 recoveries (R), and a peak NIV load of 18 patients. The top row (**A–D**) shows the effect on final cumulative outcomes, while the bottom row (**E–H**) shows the impact on peak NIV load. Each column pair examines a single parameter: (**A, E**) NIV initiation rate, α; (**B, F**) NIV recovery rate, $\theta_{0}$; (**C, G**) ICU transfer rate, γ; and (**D, H**) mortality rate, ε.

To pinpoint the most critical levers governing system load and patient outcomes, we performed a sensitivity analysis on the autonomous model, varying the four key transition rates—α (NIV initiation), $\theta_{0}$ (NIV recovery), γ (ICU transfer), and ε (mortality)—individually by ±30 % around their baseline values of α≈0.044, $\theta_{0}\approx 0.068$, γ≈0.023, and ε≈0.009 days^-1^ (Fig. 4). This baseline produced reference outcomes of approximately 13 ICU transfers, 52 mortalities, 622 recoveries, and a peak NIV load of 18 patients. The analysis revealed that while all parameters influenced outcomes, the NIV initiation rate (α) and the NIV recovery rate ($\theta_{0}$) exerted the most substantial impact on the peak operational load—that is, the maximum number of patients simultaneously requiring NIV.

The analysis of these primary load drivers confirmed their powerful, opposing effects on system strain. Increasing the NIV initiation rate (α), which represents faster patient deterioration, not only worsened downstream outcomes but also directly increased the operational burden by raising the peak NIV census from below 15 to over 19 patients (Fig. 4A, E). Conversely, enhancing the NIV recovery rate ($\theta_{0}$) delivered a substantial dual benefit: it markedly improved outcome distributions while simultaneously alleviating system strain, causing the peak NIV load to fall from over 22 to below 16 patients (Fig. 4B, F). This pronounced sensitivity is consistent with the high (68 %) recovery rate observed in our cohort and underscores that managing bed capacity is critically dependent on both the speed of patient progression towards NIV and the efficiency of their recovery from it.

In contrast, the parameters governing the specific exit pathways from the NIV state—the ICU transfer rate (γ) and the mortality rate (ε)—primarily shaped the final outcome distribution rather than driving the overall patient load. As expected, increasing γ produced a near-linear rise in cumulative ICU transfers from a baseline of Z ≈ 13, while increasing ε directly drove a substantial increase in cumulative mortality from a baseline of W ≈ 52 (Fig. 4C, D). However, these parameters had a much smaller effect on the peak NIV patient census. Variations in ε had a minimal impact on the peak load, and increasing γ even slightly decreased it, likely by clearing the NIV state more quickly (Fig. 4G, H).

Overall, these analyses provide clear and actionable insights for IRCU resource management. The system’s peak operational load—the primary constraint during a surge—is most sensitive to the throughput parameters that govern flow into and out of the NIV state (α and $\theta_{0}$). Therefore, interventions aimed at either preventing patient deterioration (addressing α) or enhancing NIV efficacy (addressing $\theta_{0}$) will have the most significant impact on managing resource strain. While the outcome parameters (γ and ε) are crucial for determining a patient’s ultimate fate (e.g., ICU use, baseline Z ≈ 13; mortality, baseline W ≈ 52), their modulation has a less pronounced effect on the immediate operational challenge of bed capacity.

### 2.3 Total occupancy modeling via LOS distribution

**Fig. 5:**
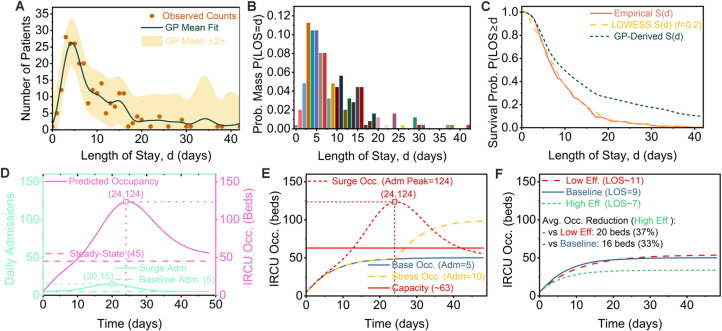
Empirical LOS distribution and its application in occupancy modelling. Panels **A–C** characterise the empirical Length-of-Stay (LOS) distribution derived from the cohort data. (**A**) The observed LOS frequency (points) is fitted with a Gaussian Process (mean: dark green line; ±2 s.d.: shaded area). (**B**) The empirical Probability Mass Function, P(LOS=d), is shown. (**C**) The empirical survival function, S(d), used for simulations, is compared with a LOWESS smoothed version (fraction f=0.2) and a GP-derived curve. Panels **D–F** apply this empirical S(d) to simulate operational scenarios. (**D**) An illustrative admission surge (from a 5/day baseline to a 15/day peak around day 20) results in a peak occupancy of ≈124 beds around day 24, far exceeding the ≈45-bed steady-state level. (**E**) Occupancy predictions for baseline (5/day), sustained stress (10/day), and surge scenarios are compared against an illustrative ≈63-bed capacity threshold. (**F**) Under a constant 5/day admission rate, varying system efficiency shows that a high-efficiency scenario (Mean LOS ≈7 d) reduces steady-state occupancy by ≈16 beds (33 %) compared to baseline (9 d); conversely, a low-efficiency scenario (Mean LOS ≈11 d) requires ≈20 beds (37 %) more than the high-efficiency case.

As a complementary approach to the state-based ODE model, we developed a convolution model to provide a direct forecast of total system load. This method is grounded in the empirical Length-of-Stay (LOS) distribution of the cohort, which was found to be a right-skewed distribution with a mean of 9.0 days and a mode within the first few days (Fig. 5A–C). From this, we derived the empirical survival function, S(d), which describes the probability of a patient remaining in the unit for at least d days. The total daily occupancy, $X_{total}(t)$, is then calculated by convolving the daily admission history, A(t), with this survival function, S(d) (see Supplementary Material for details). This data-driven approach avoids parametric assumptions and directly reflects the observed patient departure patterns.

To demonstrate the model’s utility in predicting system strain, we simulated a representative admission surge, with daily admissions rising from a baseline of 5 to a peak of 15 around day 20 (Fig. 5D). The model predicted a characteristic lagged and smoothed response, with total occupancy peaking at approximately 124 beds on day 24. This peak substantially exceeds the expected steady-state occupancy of roughly 45 beds that would result from the baseline admission rate, underscoring the severe yet delayed impact of an admission surge on bed capacity.

We further explored the operational implications for resource planning. When tested against an illustrative capacity threshold of approximately 63 beds, both a sustained stress scenario (10 admissions/day) and the surge scenario breached this limit for extended periods, highlighting the unit’s vulnerability to increased admission pressure (Fig. 5E). We also investigated the sensitivity of occupancy to system efficiency by simulating scenarios with different Mean LOS values under a constant admission rate of 5/day. Compared to the baseline Mean LOS of 9 days, a scenario simulating high efficiency (Mean LOS ≈ 7 d) yielded an average reduction of approximately 16 beds (33 %). Conversely, a scenario simulating low efficiency (Mean LOS ≈ 11 d) required an additional 20 beds (37 %) compared to the high-efficiency case (Fig. 5F).

Crucially, this convolution-based modelling complements and validates the ODE results, as both methodologies yield broadly consistent predictions of peak system load under surge conditions. The convolution model’s peak prediction of approximately 124 patients (Fig. 5D) is in close agreement with the ODE model’s peak active load of approximately 136 patients (Fig. 3L), lending significant confidence to the overall assessment of system dynamics. The two models thus offer complementary perspectives: while the ODE approach provides a mechanistic view of internal patient transitions between NIV and non-NIV states, the convolution model provides a direct, aggregate forecast of total bed demand—a key tool for operational planning.

## 3 Discussion

This study provides a critical evaluation of the clinical effectiveness and operational dynamics of a specialised IRCU during the COVID-19 pandemic. Our principal finding is that this unit, characterised by a dedicated expert staffing model, successfully managed a cohort of patients with severe respiratory failure. The most compelling evidence for this effectiveness lies in the outcomes of the highest-risk stratum: of the 77 patients requiring non-NIV, 68 % recovered entirely within the IRCU, thereby avoiding escalation to intensive care. This high success rate in a clinically challenging group underscores the unit’s capacity to function as a definitive therapeutic environment, not merely a transitional space. While the absence of mortality or ICU transfers among the 172 patients managed without NIV is a notable outcome, we interpret this primarily as confirmation of effective initial risk stratification, rather than a direct benchmark for efficacy, due to the significant and unavoidable confounding by indication. The core argument rests on the successful management of high-acuity patients, a finding that suggests the IRCU structure, including its specialised clinical teams, was instrumental in mitigating pressure on finite ICU resources during a period of immense systemic strain.

Our results resonate with, and extend, the growing body of literature confirming the strategic value of IRCUs in the pandemic response [13], [22], [23]. The overall 8 % ICU transfer rate observed in our cohort is broadly consistent with figures from other centres, although we caution that direct comparisons are complex due to underlying heterogeneity in patient populations, admission criteria, and local standards of care [22], [24]. A distinguishing feature of our study is its explicit focus on an IRCU designed with 24/7 in-house respiratory physician coverage and nursing staff who received targeted training in advanced respiratory support (Fig. 6, Fig. 7). While this observational study cannot establish definitive causality, the strong association between this specialised staffing model and the positive clinical outcomes is compelling. The high NIV success rate aligns with extensive evidence linking clinical expertise—both specialist physician involvement and higher nursing skill mix—to improved patient outcomes in acute care settings [38], [39], [40], [43]. It is highly plausible that the combination of continuous expert medical oversight and skilled nursing care synergistically enhanced the team’s capacity for effective NIV management, proactive weaning, and early recognition of deterioration, thereby contributing directly to the observed high recovery rate among these at-risk patients.

The integration of dynamic systems modelling provides a novel, mechanistic lens through which to interpret these clinical findings. Our compartmental models (Fig. 3, Fig. 4) move beyond static outcome reporting to dissect the temporal dynamics of patient flow, identifying critical leverage points for operational management. The sensitivity analysis revealed that both system load (peak NIV occupancy) and ultimate patient outcomes are most profoundly influenced by the NIV initiation rate (α) and the NIV recovery rate ($\theta_{0}$). This quantitative result provides a compelling rationale for the impact of clinical efficiency: expert-led care that optimises NIV protocols and facilitates timely recovery (increasing $\theta_{0}$) can substantially reduce adverse outcomes and alleviate resource strain. This framework offers a tangible tool for hospital administrators, enabling *in silico* evaluation of strategies, from staffing adjustments to new clinical protocols, and quantifying their potential return on investment [57], [61]. The strong concordance between our ODE model and the empirical LOS-based convolution model regarding peak occupancy under surge conditions (Section 2.3) further validates this approach for robust operational planning.

Several significant limitations must be carefully considered when interpreting our results. First, the study’s single-centre design and its focus on a specific five-month period of the COVID-19 pandemic necessarily constrain the generalisability of our findings. The dynamics observed may not directly translate to non-COVID forms of respiratory failure or to healthcare systems with different organisational structures and staffing ratios. Second, while our analysis of prospectively collected data reveals strong associations, it cannot establish causality between the specialised staffing model and the observed outcomes. Third, our modelling approach incorporates a key structural assumption—that patients in the non-NIV state experience no mortality or ICU transfers (η=0,ν=0). While empirically accurate for our specific cohort, this assumption limits the model’s direct applicability to patient populations with higher baseline acuity or different comorbidities, where adverse events may occur in less severe strata. Future modelling efforts in other contexts would need to relax this constraint. Finally, the *post hoc* sensitivity analysis indicated only moderate statistical power (63 %) to detect medium-sized effects. This constitutes a significant limitation, implying a non-trivial risk of Type II error (β≈0.37). Consequently, non-significant findings, such as the lack of gender-based differences, must be interpreted with considerable caution, as the study may have been underpowered to detect an actual underlying effect.

## 4 Conclusions

Our investigation illuminates the critical operational dynamics and clinical utility of a specialised Intermediate Respiratory Care Unit during the unprecedented challenge of the COVID-19 pandemic. We demonstrated that this newly established unit, featuring specialised physician and nursing support, successfully managed a significant cohort of patients presenting with severe COVID-19 respiratory failure. A central finding is the successful recovery of 68 % of patients requiring non-invasive ventilation entirely within the IRCU, obviating the need for ICU escalation for the majority. This outcome powerfully demonstrates the capacity of an appropriately staffed and structured intermediate care setting to function not merely as an observational waypoint, but as an active therapeutic environment capable of mitigating pressure on finite critical care resources.

These results significantly extend the growing body of evidence supporting IRCU efficacy [13], [22], [23], particularly in the pandemic context [24]. Our work distinctively foregrounds the strong association between the implementation of a specialised staffing model and these favourable outcomes, aligning with broader findings on the impact of clinical expertise and appropriate staffing levels [36], [39], [40], [43]. Furthermore, the integration of statistical analysis with dynamic systems modelling provides a novel quantitative lens through which to understand patient flow. This approach reveals the high sensitivity of system throughput and patient outcomes to parameters directly influenced by care efficiency—an efficiency plausibly enhanced by skilled multidisciplinary teams—such as the rate of NIV initiation (α) and the rate of recovery from NIV ($\theta_{0}$) (Fig. 4). This combined clinical and modelling strategy offers a more mechanistic understanding of IRCU function than previously available. Whilst illuminating, our findings open several promising avenues for future inquiry. Multi-centre prospective studies are imperative to validate our findings across diverse healthcare systems and patient populations. Delving deeper into the physiological mechanisms of NIV success [68], [69] will be crucial for refining clinical practice. The predictive power of our dynamic models could be enhanced by incorporating machine learning approaches [63], [70] to create interactive decision-support tools for operational planning. Rigorous longitudinal studies are needed to track long-term outcomes [71], and systematic evaluation of such specialised IRCUs for non-pandemic respiratory failure is essential to solidify their permanent role within the healthcare infrastructure.

## 5 Materials and methods

### 5.1 Study setting and design

We conducted this prospective cohort study at the IRCU of the University Hospital Virgen de las Nieves (UHVN), a tertiary academic medical centre in Granada, Spain. The hospital began establishing the unit in February 2020 in response to the anticipated demands of the COVID-19 pandemic (Fig. 6, point 01). We based the unit’s design on the foundational principle of a specialised clinical staffing model to ensure continuous expert coverage. Preparatory phases included targeted staff recruitment and specialised training workshops, which we delivered to the nursing contingent on advanced respiratory support modalities (Non-Invasive Ventilation [NIV] and High-Flow Nasal Oxygen [HFNO]) starting in December 2020 (Fig. 6, point 02).

The Spanish Society of Pneumology and Thoracic Surgery (SEPAR) formally accredited the IRCU at its highest level of complexity, and the unit became operational in April 2021 (Fig. 6, point 03). We staffed the unit with dedicated respiratory physicians who provided 24/7 in-house coverage, supported by specialised nursing personnel, and we equipped it comprehensively with advanced monitoring technology. The Provincial Research Ethics Committee of Granada (CEIM/CEI Provincial de Granada) granted ethical approval for the overarching project, which encompassed the data collection for this analysis, on 24 June 2020. In accordance with the Declaration of Helsinki and this approval, we obtained written informed consent from all participants or their legally authorised representatives. We enrolled patients for the analysis presented herein from April 2021 to August 2021 (Fig. 6, points 03–04).

### 5.2 Study population

We included in this study adult patients (age ≥ 18 years) admitted to the UHVN IRCU between April and August 2021 (Fig. 6, points 03–04). We established the primary admission criterion as severe respiratory failure, predominantly associated with confirmed SARS-CoV-2 infection and compatible radiological findings. Accordingly, we excluded patients admitted principally for non-pulmonary primary diagnoses. Although the clinical team considered other potential exclusion criteria in individual patient management, such as pre-existing directives limiting therapeutic escalation (e.g., ’do not resuscitate’ orders), we did not systematically apply these as absolute exclusion criteria for cohort enrolment. The final analytic sample comprised 249 consecutive patients who received care in the IRCU during the defined study interval (Fig. 1, Fig. 2).

### 5.3 Clinical management and data collection

Upon each patient’s admission to the IRCU, our dedicated medical and nursing staff conducted a standardised initial assessment protocol (Fig. 7, Reception & Evaluation phases), which included vital sign measurement and a comprehensive clinical evaluation. The clinical team initiated NIV or HFNO based on collaborative clinical judgement for patients who met the criteria for severe respiratory failure (typically a fractional inspired oxygen [FiO2] requirement exceeding 40 %) (Fig. 7, Treatment phase). The principal respiratory support modalities we employed included Continuous Positive Airway Pressure (CPAP) [7], [13], [22], Bilevel Positive Airway Pressure (BiPAP) with Respironics V60 ventilators (Philips) [11], [13], and HFNO [12], [13]. Our use of HFNO adhered to evolving international guidance during the pandemic, incorporating WHO recommendations for vigilant monitoring [72], [73]. To ensure confidentiality as per our ethical approval, the research team de-identified all patient data prior to analysis.

Continuous cardiorespiratory monitoring informed ongoing clinical management. A key institutional guideline mandated proactive multidisciplinary consultation, particularly with the ICU team, for patients exhibiting sustained high oxygen requirements (e.g., FiO2 > 80 % despite NIV optimisation) to facilitate timely decisions regarding the appropriate level of care [7], [11]. This collaborative framework guided the determination of each patient’s trajectory (Fig. 7, Development phase), whether ICU escalation, transfer to a general medical ward upon improvement, or direct discharge home. Throughout each IRCU admission, we maintained established nurse-to-patient (approximately 1:4) and physician-to-patient (approximately 1:6) ratios, ensuring continuous specialised clinical oversight. We collected data prospectively using standardised forms, capturing patient demographics, pertinent comorbidities, respiratory support details, length of stay (LOS), and definitive IRCU outcomes (ICU transfer, in-IRCU mortality, or recovery), which constituted the dataset for our subsequent analysis (Fig. 1, Fig. 2).

### 5.4 Statistical analysis and visualization

We employed descriptive statistics to summarise the cohort’s baseline characteristics and clinical outcomes, reporting counts (N), percentages (%), medians, and interquartile ranges (IQR). We visualised the distributions of continuous variables using violin plots (Fig. 1). For intergroup comparisons, we used the Mann-Whitney U test for continuous variables (e.g., Figs. 1A and 2A) and the Chi-squared or Fisher’s exact test, as appropriate, for categorical variables. To evaluate associations between predictor variables (age, gender, NIV status) and binary clinical outcomes (NIV use, ICU transfer, mortality), we calculated both crude Risk Ratios (RR) with 95 % confidence intervals (CI) (Fig. 2D) and fitted multivariable logistic regression models. These models yielded adjusted OR with 95 % CIs (Fig. 2C), allowing us to control for potential confounding by patient age and gender where applicable. We established statistical significance at a two-tailed p-value threshold of < 0.05.

We characterised the distribution of total Length-of-Stay (LOS) empirically using Probability Mass Functions (PMF) and Kaplan-Meier-derived Survival Functions (S(d)) (Fig. 5). To generate smoothed estimates, we also fitted a Gaussian Process (GP) regression model [70] to the observed LOS frequency data (Fig. 5A). We selected this non-parametric Bayesian technique to address the reviewer’s concern regarding model choice; its flexibility allows for modelling complex, real-world distributions without imposing the strong assumptions of standard parametric survival models (e.g., Weibull, log-normal), thereby providing a more robust, data-driven characterisation. However, for the occupancy simulations, we primarily utilised the directly calculated empirical S(d) to ground our forecasts in the observed data. We performed all statistical computations using Python (version 3.7; libraries: SciPy, Statsmodels, Matplotlib, Scikit-learn) and R (version 4.3.2; ‘pwr‘ package). We prepared all figures, including diagrams (Fig. 6, Fig. 7) and schematics, using Inkscape (version 1.3) [74]. Given that missing data were minimal (<1 %), we conducted a complete case analysis.

To address the study’s statistical power, as highlighted by the reviewer, we conducted a *post hoc sensitivity analysis* using G*Power (version 3.1) [75]. We emphasise that this analysis evaluates the study’s sensitivity to detect a pre-specified, conventional effect size, rather than the power based on observed effects. Specifically, for our final sample sizes (Total N=249; Non-NIV $n_{1}=172$, NIV $n_{2}=77$), we calculated the achieved power (1-β) to detect a ’medium’ effect size (Cohen’s d = 0.5) [76] between groups using a two-tailed independent samples *t*-test framework at an alpha (α) of 0.05. The resulting achieved power was approximately **0.63 (63 %)**. This moderate statistical power indicates a non-trivial risk of a Type II error (β≈0.37), suggesting that we may have failed to detect an actual medium-sized effect if one existed. We therefore urge considerable caution in interpreting non-significant findings, particularly for secondary comparisons, and underscore the need for larger studies to investigate more subtle differences.

## CRediT authorship contribution statement

**Ana Carmen Navas-Ortega:** Writing – review & editing, Writing – original draft, Visualization, Methodology, Investigation, Data curation, Conceptualization. **José Antonio Sánchez-Martínez:** Writing - review & editing, Writing – original draft, Validation, Methodology, Investigation, Data curation, Conceptualization. **Paula García-Flores:** Writing – review & editing, Writing – original draft, Methodology, Investigation, Data curation. **Concepción Morales-García:** Writing – review & editing, Writing – original draft, Validation, Supervision, Methodology, Investigation, Conceptualization. **Rene Fabregas:** Writing – review & editing, Writing – original draft, Visualization, Validation, Supervision, Methodology, Investigation, Formal analysis, Data curation, Conceptualization.

## Ethics approval

The study adhered to the Declaration of Helsinki. Ethical approval for the broader project encompassing the collection of data analysed in this manuscript was provided by the *Provincial Research Ethics Committee of Granada (CEIM/CEI Provincial de Granada)* on June 24, 2020. Written informed consent was obtained from all participants or their legal representatives prior to inclusion, following the stipulations of the approved protocol. Participant data confidentiality was maintained throughout the study.

## Declaration of competing interest

The authors affirm that there are no competing interests—financial, personal, or otherwise—that have influenced, or could be perceived to have influenced, the conduct or reporting of the research described in this paper.

## Data Availability

The Python scripts used for the ODE modelling (Fig. 3, Fig. 4), Length-of-Stay analysis, and occupancy simulations (Fig. 5), along with the necessary input data files (“patient_data.csv”, “ircu_data.csv”), are publicly available. The specific version of the code and data corresponding to the results presented in this manuscript (Version v1.0.0) has been archived on Zenodo and can be accessed via the following DOI: https://doi.org/10.5281/zenodo.15286823. The development repository is hosted on GitHub at https://github.com/renee29/IRCU_Patient_Flow_Modeling_Scripts.
