# Supplementary material for: Data-driven modelling of IRCU patient flow during the COVID-19 pandemic
Source: Comput Struct Biotechnol J. 2025 Oct 17;27:4657–67. doi: 10.1016/j.csbj.2025.10.017 (PMC12613035; doi:10.1016/j.csbj.2025.10.017)
Supplement: Multimedia Component 1 [file mmc1.pdf]

Supplementary Material: Data-Driven Modelling of IRCU Patient Flow during the COVID-19 Pandemic

Ana Carmen Navas-Ortega[‡1], José Antonio Sánchez-Martínez[‡1,2], Paula García-Flores[1,2], Concepción Morales-García[1,2], and Rene Fabregas[3]

[1]Department of Pneumology, University Hospital Virgen de Las Nieves, Granada, Spain.
[2]Biosanitary Research Institute of Granada-Ibs, Granada, Spain.
[3]Department of Applied Mathematics and Modeling Nature (MNat) Research Unit, Faculty of Sciences, University of Granada, Granada, Spain.
Corresponding Author: Concepción Morales-García, `concepcion.morales.sspa@juntadeandalucia.es` and Rene Fabregas, `rfabregas@ugr.es`.

Tuesday 7th October, 2025

S1 Discrete Model of IRCU with Intervention Modulation.

Effective management of Intermediate Respiratory Care Units (IRCUs) requires a quantitative understanding of patient flow dynamics, particularly under varying admission loads and evolving clinical interventions. This section introduces a discrete-time compartmental model specifically designed to capture patient progression within an IRCU, explicitly accounting for the use of non-invasive ventilation (NIV) and the impact of targeted clinical management strategies. By subdividing the IRCU population based on NIV status (state X for non-NIV, state Y for active NIV), the model utilizes a system of difference equations to characterize patient trajectories including admission, NIV initiation, recovery pathways, transfer to the Intensive Care Unit (ICU), and mortality (exitus), focusing on flows originating within the IRCU environment. Crucially, the model integrates parameters representing key clinical interventions aimed at modulating patient outcomes, providing a framework for evaluating their effectiveness.

We define the state variables at discrete time t (typically days) as follows:

- $X(t)$: Number of patients within the IRCU *not* receiving NIV at time t. This includes newly admitted patients or those under standard observation/care. [patients].
- $Y(t)$: Number of patients within the IRCU *actively receiving* NIV therapy at time t. [patients].
- $Z(t)$: Cumulative number of patients transferred *from* the IRCU pathway *to* the ICU up to time t. [patients].
- $W(t)$: Cumulative number of patients experiencing exitus *within* the IRCU pathway (either from state X or Y) up to time t. [patients].
- $R(t)$: Cumulative number of patients designated as recovered (discharged home or transferred to a general ward) *from* the IRCU pathway up to time t. [patients].

Let $A(t)$ denote the influx of new patient admissions directed to the IRCU system during the time interval $[t, t+1)$ [patients/time step]. The model dynamics are governed by baseline transition parameters (fractional rates or probabilities per time step) and specific intervention modulation parameters:

- **Baseline Transitions:** α (rate of NIV initiation for patients in X), γ (baseline transition rate from NIV (Y) to ICU), ε (baseline exitus rate directly from NIV (Y)), θ_0 (baseline recovery rate directly from NIV (Y)), ρ (direct recovery/discharge rate from non-NIV state (X)), η (direct ICU transfer rate from non-NIV state (X)), ν (direct exitus rate from non-NIV state (X)).

‡ These authors contributed equally.

- **Intervention Modulators:** These dimensionless factors ($0 \leq \gamma_0, \varepsilon_0 < 1$; $\Delta\theta, \lambda \geq 0$) represent the impact of specific clinical strategies: ε_0: Relative reduction in NIV mortality rate due to protocols like enhanced monitoring or staff training [1, 2], resulting in an effective rate $\varepsilon_{eff} = \varepsilon(1 - \varepsilon_0)$. γ_0: Relative reduction in NIV-to-ICU transfers achieved through optimized NIV management or timely intervention protocols [3, 4], leading to an effective rate $\gamma_{eff} = \gamma(1 - \gamma_0)$. $\Delta\theta, \lambda$: Parameters governing transient improvements in the NIV recovery rate, modeling effects like temporary staffing surges [5, 6]. The effective recovery rate becomes time-dependent: $\theta(t) = \theta_0 + \Delta\theta e^{-\lambda t}$, where $\Delta\theta$ is the initial boost magnitude and λ is the decay rate of the effect.

(Note: Dynamics post-ICU transfer, such as ICU length of stay or outcomes from the ICU, are considered outside the scope of this IRCU-focused model).

The structure of patient flow, incorporating the points of intervention, is illustrated in Figure S1. Admissions $A(t)$ feed into the non-NIV state $X(t)$. Patients in $X(t)$ can recover directly (R), be transferred to ICU (Z), experience exitus (W), or initiate NIV therapy, moving to state $Y(t)$. From the NIV state $Y(t)$, patients can recover (R), be transferred to ICU (Z), or experience exitus (W). The intervention modulators ($\gamma_0, \varepsilon_0, \Delta\theta, \lambda$) specifically act upon the transition rates originating from the active NIV state $Y(t)$, reflecting targeted efforts to improve outcomes for this high-acuity patient group. Compartmental diagrams of this nature are standard tools for visualizing system dynamics in mathematical biology and healthcare modeling [7, 8, 9].

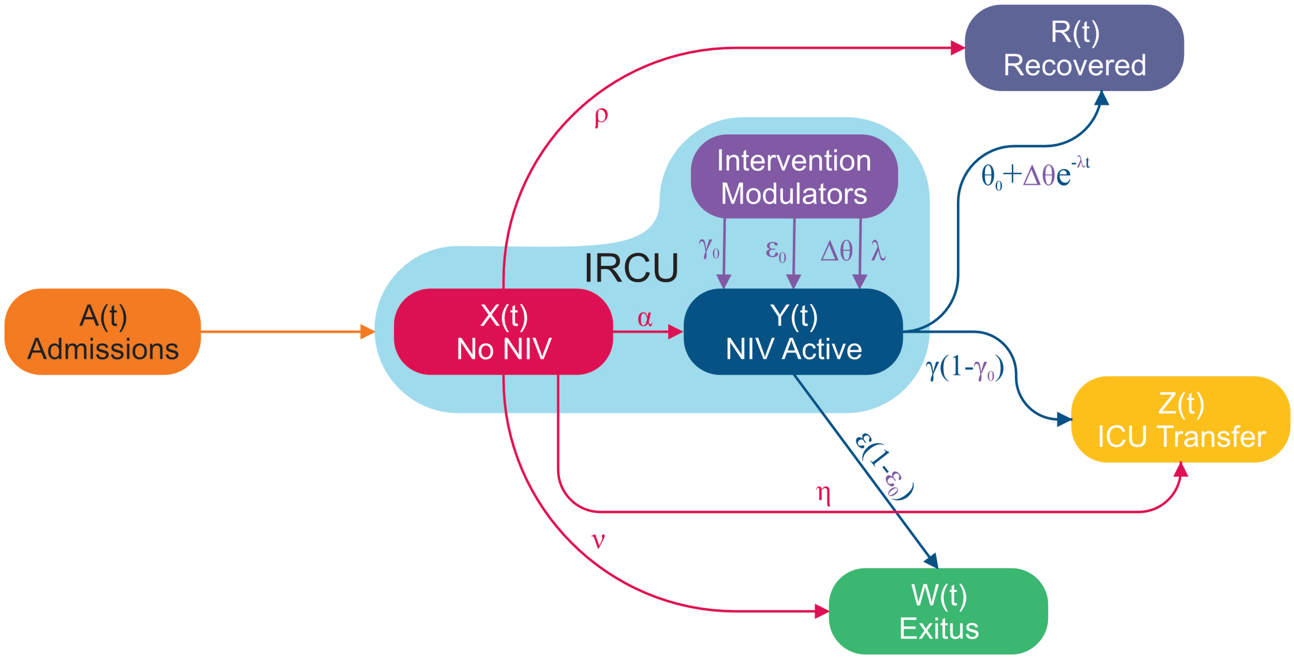

Figure S1| Integrated Compartmental Model of IRCU Patient Flow with Intervention Pathways. Nodes represent patient states: Admissions ($A(t)$), patients within the IRCU distinguished by NIV status ($X(t)$: no NIV; $Y(t)$: active NIV, highlighted within the IRCU region), and cumulative outcomes ($R(t)$: recovered, $Z(t)$: ICU transfer, $W(t)$: exitus). Solid arrows depict patient flow rates. Parameters α, ρ, η, ν govern transitions from state X. Parameters $\gamma, \varepsilon, \theta_0$ represent baseline transition rates from the active NIV state Y. The 'Intervention Modulators' ($\gamma_0, \varepsilon_0, \Delta\theta, \lambda$, depicted in the pink box) quantify the impact of clinical strategies by modifying the effective rates of ICU transfer ($\gamma(1 - \gamma_0)$), exitus ($\varepsilon(1 - \varepsilon_0)$), and recovery ($\theta(t) = \theta_0 + \Delta\theta e^{-\lambda t}$) from state Y, with conceptual links indicated by faded arrows.

The mathematical formulation translates the flows depicted in Figure S1 into a system of first-order difference equations. Each term corresponds to an inflow or outflow from a compartment,

incorporating the relevant baseline rates and intervention modulators:

$$X(t+1) = X(t) + A(t) - (\alpha + \rho + \eta + \nu)X(t) \tag{S1}$$

$$Y(t+1) = Y(t) + \alpha X(t) - \left[\gamma(1-\gamma_0) + \underbrace{\left(\theta_0 + \Delta\theta e^{-\lambda t}\right)}_{\theta(t)} + \varepsilon(1-\varepsilon_0) \right] Y(t) \tag{S2}$$

$$Z(t+1) = Z(t) + \gamma(1-\gamma_0)Y(t) + \eta X(t) \tag{S3}$$

$$W(t+1) = W(t) + \varepsilon(1-\varepsilon_0)Y(t) + \nu X(t) \tag{S4}$$

$$R(t+1) = R(t) + \left[\theta_0 + \Delta\theta e^{-\lambda t}\right] Y(t) + \rho X(t) \tag{S5}$$

Equation (S1) describes the change in the non-NIV population (X), balancing admissions $A(t)$ against outflows due to NIV initiation (α), direct recovery (ρ), direct ICU transfer (η), and direct exitus (ν). Equation (S2) tracks the active NIV population (Y), with inflow from state X and outflows to ICU, exitus, and recovery, where these rates are potentially modified by interventions ($\gamma_0, \varepsilon_0, \Delta\theta, \lambda$). Equations (S3), (S4), and (S5) accumulate patients transitioning into the ICU, exitus, and recovered states, respectively, incorporating contributions from both X and the modulated Y pathways.

This model structure aligns with observed clinical workflows in intermediate care settings [10, 11] and adheres to established principles of compartmental modeling in mathematical biology and epidemiology [12, 13]. Its explicit incorporation of intervention parameters enhances its utility for healthcare operational analysis. The framework allows for quantitative exploration of various clinical scenarios:

- Evaluating the impact of quality improvement initiatives targeting specific outcomes (e.g., reducing NIV-associated mortality via ε_0).
- Assessing the benefits of strategies aimed at preventing deterioration (e.g., reducing ICU transfers via γ_0).
- Simulating the dynamic effects of resource adjustments, such as temporary staffing increases ($\Delta\theta, \lambda$).
- Investigating potential non-linearities or threshold effects in intervention efficacy.
- Exploring synergies or trade-offs between simultaneously implemented interventions.

Such compartmental models are foundational for understanding population dynamics [7, 8, 14] and provide critical tools for healthcare planning, resource allocation, and system optimization [15, 16]. Furthermore, the deterministic core can be integrated with data-driven approaches (e.g., Bayesian inference, machine learning) for parameter estimation, uncertainty quantification, and real-time adaptation [9, 17], enhancing predictive accuracy. Consequently, this model serves as a robust computational platform—a form of "digital twin"—for the *in silico* evaluation of operational policies and clinical protocols, facilitating evidence-informed decision-making to improve patient outcomes and system efficiency within the IRCU [6].

S2 Transformation to a Continuous-Time Framework.

While the discrete-time model provides valuable insights into patient flow dynamics at specific intervals, transitioning to a continuous-time framework offers complementary advantages. Continuous models, expressed as systems of ordinary differential equations (ODEs), allow for the application of calculus-based analytical techniques and can represent underlying processes that evolve smoothly over time. This section details the derivation of such a continuous model from our discrete formulation.

Recall the discrete model incorporates patient movements between states during each time interval Δt. Considering small intervals, we approximate the change in patient numbers using instantaneous rates. Let $A(t)$ be the rate of new admissions [patients/time] and α, ρ, γ, etc., be instantaneous transition rates [1/time]. The net change in a compartment f over Δt is approximately the sum of

inflows minus outflows:

$$\begin{aligned}
X(t+\Delta t) - X(t) &\approx [A(t) - (\alpha+\rho+\eta+\nu)X(t)]\Delta t,\\
Y(t+\Delta t) - Y(t) &\approx [\alpha X(t) - (\gamma(1-\gamma_0) + \theta(t) + \varepsilon(1-\varepsilon_0))Y(t)]\Delta t,\\
Z(t+\Delta t) - Z(t) &\approx [\gamma(1-\gamma_0)Y(t) + \eta X(t)]\Delta t,\\
W(t+\Delta t) - W(t) &\approx [\varepsilon(1-\varepsilon_0)Y(t) + \nu X(t)]\Delta t,\\
R(t+\Delta t) - R(t) &\approx [\theta(t)Y(t) + \rho X(t)]\Delta t.
\end{aligned}$$

Using the Taylor expansion $f(t+\Delta t) \approx f(t) + \frac{df}{dt}\Delta t$ and taking the limit as $\Delta t \to 0$, we derive the continuous-time ODE system:

$$\frac{dX}{dt} = A(t) - (\alpha+\rho+\eta+\nu)X(t), \tag{S6}$$

$$\frac{dY}{dt} = \alpha X(t) - [\gamma(1-\gamma_0) + \theta(t) + \varepsilon(1-\varepsilon_0)]Y(t), \tag{S7}$$

$$\frac{dZ}{dt} = \gamma(1-\gamma_0)Y(t) + \eta X(t), \tag{S8}$$

$$\frac{dW}{dt} = \varepsilon(1-\varepsilon_0)Y(t) + \nu X(t), \tag{S9}$$

$$\frac{dR}{dt} = \theta(t)Y(t) + \rho X(t). \tag{S10}$$

This continuous system explicitly incorporates the clinical interventions: **Mortality Reduction (ε_0):** Scaled rate $\varepsilon(1-\varepsilon_0)$ reflects mitigation from enhanced protocols [1]. **ICU Transfer Mitigation (γ_0):** Reduced rate $\gamma(1-\gamma_0)$ models optimized NIV management preventing ICU transfers [4]. **Transient Staffing Effects ($\Delta\theta, \lambda$):** Time-dependent recovery $\theta(t) = \theta_0 + \Delta\theta e^{-\lambda t}$ represents enhanced potential during staffing surges [5, 6]. The inclusion of $\theta(t)$ renders the system non-autonomous.

S2.1 Scaling and Non-dimensionalization.

To understand core dynamics, we employ dimensional analysis. We define characteristic scales: $\tau = (\alpha+\rho+\eta+\nu)^{-1}$ (characteristic time in state X) and $N_0 = A_0\tau$ (characteristic population size, with A_0 a baseline admission rate).

Dimensionless variables $(\tilde{t}, \tilde{X}, \tilde{Y}, \tilde{Z}, \tilde{W}, \tilde{R}, \tilde{A})$ and parameters (π) are introduced:

$$\tilde{t} = t/\tau, \quad \tilde{X} = X/N_0, \quad \ldots \quad \tilde{A}(\tilde{t}) = A(t)/A_0$$

$$\begin{aligned}
&\pi_\alpha = \alpha\tau, \quad \pi_\rho = \rho\tau, \quad \pi_\eta = \eta\tau, \quad \pi_\nu = \nu\tau, \quad (\text{Note: } \pi_\alpha + \pi_\rho + \pi_\eta + \pi_\nu = 1)\\
&\pi_\gamma = \gamma\tau, \quad \pi_\varepsilon = \varepsilon\tau, \quad \pi_\theta(\tilde{t}) = \theta(t)\tau, \quad \pi_\lambda = \lambda\tau
\end{aligned}$$

Substituting these yields the dimensionless system:

$$\frac{d\tilde{X}}{d\tilde{t}} = \tilde{A}(\tilde{t}) - \tilde{X}, \tag{S11}$$

$$\frac{d\tilde{Y}}{d\tilde{t}} = \pi_\alpha\tilde{X} - [\pi_\gamma(1-\gamma_0) + \pi_\theta(\tilde{t}) + \pi_\varepsilon(1-\varepsilon_0)]\,\tilde{Y}, \tag{S12}$$

$$\frac{d\tilde{Z}}{d\tilde{t}} = \pi_\gamma(1-\gamma_0)\tilde{Y} + \pi_\eta\tilde{X}, \tag{S13}$$

$$\frac{d\tilde{W}}{d\tilde{t}} = \pi_\varepsilon(1-\varepsilon_0)\tilde{Y} + \pi_\nu\tilde{X}, \tag{S14}$$

$$\frac{d\tilde{R}}{d\tilde{t}} = \pi_\theta(\tilde{t})\tilde{Y} + \pi_\rho\tilde{X}. \tag{S15}$$

where $\pi_\theta(\tilde{t}) = \pi_{\theta_0} + \pi_{\Delta\theta}e^{-\pi_\lambda\tilde{t}}$.

S2.2 Analytical Solution for the Autonomous Case.

While the general non-autonomous system ((S11)–(S15)) typically requires numerical integration due to the time-dependent coefficient $\pi_\theta(\tilde{t})$, an exact analytical solution can be derived for the important

special case where the system is autonomous. This occurs when the transient staffing effect is absent ($\pi_{\Delta\theta} = 0$, thus $\pi_\theta(\tilde{t}) = \pi_{\theta_0} =$ constant) and the admission rate is constant ($\tilde{A}(\tilde{t}) = \tilde{A}$). Assuming zero initial conditions ($\tilde{X}(0) = \tilde{Y}(0) = \tilde{Z}(0) = \tilde{W}(0) = \tilde{R}(0) = 0$), the solution unfolds sequentially.

First, the equation for $\tilde{X}$ (S11) yields:

$$\tilde{X}(t) = \tilde{A}(1 - e^{-t}).$$

Next, defining the constant total outflow rate from state Y as $K_1 = \pi_\gamma(1-\gamma_0) + \pi_{\theta_0} + \pi_\varepsilon(1-\varepsilon_0)$, the equation for $\tilde{Y}$ (S12) becomes a standard linear first-order ODE with constant coefficients forced by $\tilde{X}(t)$. Assuming $K_1 \neq 1$ and $K_1 \neq 0$, its solution is:

$$\tilde{Y}(t) = \frac{\pi_\alpha \tilde{A}}{K_1(K_1-1)} \left[(K_1 - 1) - K_1 e^{-t} + e^{-K_1 t}\right].$$

(In the specific case where $K_1 = 1$, the solution takes the form $\tilde{Y}(t) = \pi_\alpha \tilde{A}(1 - e^{-t} - te^{-t})$).

Finally, the cumulative variables $\tilde{Z}(t)$, $\tilde{W}(t)$, and $\tilde{R}(t)$ are obtained by direct integration of their respective differential equations (S13), (S14), and (S15), substituting the derived expressions for $\tilde{X}(t)$ and $\tilde{Y}(t)$. The resulting solutions are linear combinations of a term linear in time t, a constant term, and exponential terms e^{-t} and $e^{-K_1 t}$. For instance, the solution for cumulative ICU transfers $\tilde{Z}(t)$ takes the form:

$$\tilde{Z}(t) = A_Z t + B'_Z(1 - e^{-t}) + C'_Z(1 - e^{-K_1 t}),$$

where $A_Z = \tilde{A}(\pi_{\gamma'}\pi_\alpha/K_1 + \pi_\eta)$ is the asymptotic accumulation rate (with $\pi_{\gamma'} = \pi_\gamma(1-\gamma_0)$), and B'_Z, C'_Z are constants derived from the integration involving B_Z and C_Z as defined in the detailed derivation. Analogous expressions hold for $\tilde{W}(t)$ and $\tilde{R}(t)$. This analytical solution for the autonomous case provides a valuable baseline for understanding the system's fundamental dynamics and validating numerical solvers used for the more general non-autonomous system.

S3 Steady State, Sensitivity, and Simulated Interventions.

We analyze the system's behavior under two conditions: the autonomous case, representing baseline operations with constant parameters, and the non-autonomous case, incorporating time-dependent interventions like staffing surges.

Autonomous Case: Steady-State Equilibrium ($\pi_\theta(t) = \pi_{\theta_0}$).

First, consider the system under constant baseline conditions, where the admission rate is constant ($\tilde{A}(\tilde{t}) = 1$) and the recovery rate from NIV is also constant ($\pi_\theta(\tilde{t}) = \pi_{\theta_0}$, i.e., $\pi_{\Delta\theta} = 0$). In this autonomous scenario, we can determine the equilibrium points (steady states) by setting the time derivatives in Equations (S11) and (S12) to zero. This yields the steady-state populations for the IRCU compartments:

$$\tilde{X}^* = 1, \quad \tilde{Y}^* = \frac{\pi_\alpha}{\pi_\gamma(1-\gamma_0) + \pi_{\theta_0} + \pi_\varepsilon(1-\varepsilon_0)}.$$

The steady-state occupancy levels depend directly on the relative transition rates (π values) and the baseline intervention levels (γ_0, ε_0). A standard stability analysis of the Jacobian matrix for the $(\tilde{X}, \tilde{Y})$ subsystem confirms that this equilibrium $(\tilde{X}^*, \tilde{Y}^*)$ is locally asymptotically stable for all physically realistic (non-negative) parameter values.

For the cumulative compartments $(\tilde{Z}, \tilde{W}, \tilde{R})$, their governing equations involve only non-negative inflow terms when evaluated at the steady state $(\tilde{X}^*, \tilde{Y}^*)$. For instance, the rate of change for cumulative ICU transfers becomes:

$$\left(\frac{d\tilde{Z}}{d\tilde{t}}\right)^* = \pi_\gamma(1-\gamma_0)\tilde{Y}^* + \pi_\eta \tilde{X}^* = \frac{\pi_\gamma(1-\gamma_0)\pi_\alpha}{\pi_\gamma(1-\gamma_0) + \pi_{\theta_0} + \pi_\varepsilon(1-\varepsilon_0)} + \pi_\eta.$$

Since this rate is constant and positive, the cumulative states $\tilde{Z}(t)$, $\tilde{W}(t)$, and $\tilde{R}(t)$ do not reach a finite steady state but instead grow linearly over time at constant rates determined by the steady-state populations $\tilde{X}^*$ and $\tilde{Y}^*$. The primary operational concern is therefore not instability, but whether these constant accumulation rates (particularly for $\tilde{Z}$) lead to exceeding downstream capacities (like ICU beds) over extended periods.

Non-Autonomous Case: Asymptotic Behavior ($\pi_\theta(t) = \pi_{\theta_0} + \pi_{\Delta\theta} e^{-\pi_\lambda \tilde{t}}$).

When interventions introduce time-dependency, such as the transient staffing surge modeled by $\pi_\theta(t)$ with $\pi_{\Delta\theta} > 0$ and $\pi_\lambda > 0$, the system becomes non-autonomous. By definition, such systems do not possess fixed steady-state equilibrium points while the parameters are changing. The system's state ($\tilde{X}(t), \tilde{Y}(t)$, etc.) evolves dynamically according to the time-varying recovery rate.

However, we can analyze the long-term (asymptotic) behavior. As $\tilde{t} \to \infty$, the transient term $\pi_{\Delta\theta} e^{-\pi_\lambda \tilde{t}}$ decays to zero. Consequently, $\pi_\theta(t) \to \pi_{\theta_0}$. This means that the non-autonomous system asymptotically approaches the behavior of the autonomous system discussed above. The state variables $\tilde{X}(t)$ and $\tilde{Y}(t)$ will converge towards the steady-state values $\tilde{X}^*$ and $\tilde{Y}^*$ calculated using the baseline recovery rate π_{θ_0}. Similarly, the *rates* of accumulation for $\tilde{Z}(t)$, $\tilde{W}(t)$, and $\tilde{R}(t)$ will asymptotically approach the constant rates derived for the autonomous case.

Therefore, while the system exhibits transient dynamics during the period where the staffing surge effect is significant, its long-term equilibrium behavior is governed by the baseline parameters. The primary impact of the transient intervention ($\pi_{\Delta\theta}, \pi_\lambda$) lies in altering the *trajectory* towards this asymptotic state, potentially mitigating peak loads or accelerating recovery during critical periods, as explored in the numerical simulations (Section S3.2).

S3.1 Parametric Sensitivity at Equilibrium: Identifying Key Levers.

To understand which parameters most significantly influence the system's long-term behavior, we perform a parametric sensitivity analysis. This analysis focuses on the steady-state Non-Invasive Ventilation (NIV) occupancy ($\tilde{Y}^*$) derived from the **autonomous system** (or, equivalently, the asymptotic state approached by the non-autonomous system as $t \to \infty$). We quantify the influence of each parameter p using dimensionless relative sensitivity indices, defined as $S_p = \frac{\partial \tilde{Y}^*}{\partial p} \cdot \frac{p}{\tilde{Y}^*}$. This index measures the proportional change in $\tilde{Y}^*$ resulting from a proportional change in the parameter p. A positive index indicates that increasing the parameter increases $\tilde{Y}^*$, while a negative index signifies the opposite relationship. This analysis helps identify the most effective levers for managing NIV resource utilization within the IRCU.

Sensitivity to NIV Admission Rate (π_α). The sensitivity of the equilibrium NIV occupancy to the relative rate at which non-NIV patients transition to requiring NIV is calculated as:

$$S_{\pi_\alpha} = \frac{\partial \tilde{Y}^*}{\partial \pi_\alpha} \cdot \frac{\pi_\alpha}{\tilde{Y}^*} = 1$$

This index value of exactly 1 signifies a direct, linear, and proportional relationship. A 10% increase in π_α (representing, for example, an influx of patients with higher acuity requiring NIV sooner) leads directly to a 10% increase in the long-term equilibrium occupancy of NIV beds ($\tilde{Y}^*$). This highlights the system's direct vulnerability to changes in the clinical characteristics of incoming patients or factors that accelerate the need for NIV initiation. Managing this sensitivity relies primarily on external factors influencing admission acuity or internal processes affecting the α transition, rather than downstream interventions from state Y.

Sensitivity to Baseline NIV Recovery Rate (π_{θ_0}). The influence of the baseline rate at which patients recover directly from NIV is given by:

$$S_{\pi_{\theta_0}} = \frac{\partial \tilde{Y}^*}{\partial \pi_{\theta_0}} \cdot \frac{\pi_{\theta_0}}{\tilde{Y}^*} = \frac{-\pi_{\theta_0}}{\pi_\gamma(1-\gamma_0) + \pi_{\theta_0} + \pi_\varepsilon(1-\varepsilon_0)}$$

This sensitivity index is inherently negative ($S_{\pi_{\theta_0}} < 0$), indicating that enhancing the baseline recovery rate from NIV (π_{θ_0}) serves to decrease the long-term occupancy $\tilde{Y}^*$. The magnitude of this effect is determined by the proportion that baseline recovery contributes to the total outflow rate from the NIV state (Y). Interventions aimed at improving general NIV care efficacy, shortening duration of NIV dependency, or accelerating recovery would leverage this sensitivity to reduce the equilibrium burden on NIV resources.

Sensitivity to Mitigated ICU Transfer Rate ($\pi_\gamma(1-\gamma_0)$). The impact of the rate at which NIV patients deteriorate and require transfer to the ICU, considering mitigation efforts (γ_0), is:

$$S_{\pi_\gamma(1-\gamma_0)} = \frac{\partial \tilde{Y}^*}{\partial(\pi_\gamma(1-\gamma_0))} \cdot \frac{\pi_\gamma(1-\gamma_0)}{\tilde{Y}^*} = \frac{-\pi_\gamma(1-\gamma_0)}{\pi_\gamma(1-\gamma_0)+\pi_{\theta_0}+\pi_\varepsilon(1-\varepsilon_0)}$$

This index is also negative ($S_{\pi_\gamma(1-\gamma_0)} < 0$). Increasing the effectiveness of protocols designed to prevent avoidable ICU transfers (i.e., increasing γ_0, which decreases the overall term $\pi_\gamma(1-\gamma_0)$) leads to a reduction in the equilibrium NIV occupancy $\tilde{Y}^*$. The magnitude of this sensitivity depends on the relative importance of the ICU transfer pathway compared to recovery and exitus pathways from state Y. If ICU transfer is a dominant pathway (large π_γ), interventions targeting γ_0 can be particularly impactful levers for managing $\tilde{Y}^*$.

Sensitivity to Mitigated NIV Exitus Rate ($\pi_\varepsilon(1-\varepsilon_0)$). Finally, the sensitivity to the rate of mortality occurring directly from the NIV state, accounting for mortality reduction interventions (ε_0), is:

$$S_{\pi_\varepsilon(1-\varepsilon_0)} = \frac{\partial \tilde{Y}^*}{\partial(\pi_\varepsilon(1-\varepsilon_0))} \cdot \frac{\pi_\varepsilon(1-\varepsilon_0)}{\tilde{Y}^*} = \frac{-\pi_\varepsilon(1-\varepsilon_0)}{\pi_\gamma(1-\gamma_0)+\pi_{\theta_0}+\pi_\varepsilon(1-\varepsilon_0)}$$

Similar to recovery and ICU transfer, this sensitivity is negative ($S_{\pi_\varepsilon(1-\varepsilon_0)} < 0$). Implementing protocols or enhancing care to reduce mortality while on NIV (i.e., increasing ε_0, thus decreasing the term $\pi_\varepsilon(1-\varepsilon_0)$) contributes to lowering the long-term NIV occupancy $\tilde{Y}^*$, as fewer patients remain in state Y prior to death. The relative impact compared to modifying recovery or ICU transfer rates depends on the baseline mortality rate π_ε and the effectiveness of the reduction intervention ε_0.

It must be emphasized that these sensitivity indices quantify the impact of parameter variations on the *equilibrium* state $\tilde{Y}^*$ of the autonomous system, or equivalently, the *asymptotic* state approached by the non-autonomous system long after transient effects have subsided. During periods where time-dependent interventions are active (specifically, when the staffing surge term $\pi_{\Delta\theta}e^{-\pi_\lambda \tilde{t}}$ is non-negligible), the instantaneous sensitivity of the system state (e.g., $\tilde{Y}(t)$) to parameter changes will differ from these calculated equilibrium sensitivities. The parameters governing the transient enhancement ($\pi_{\Delta\theta}, \pi_\lambda$) directly shape the system's trajectory during that phase but do not influence the final asymptotic state $\tilde{Y}^*$ upon which this sensitivity analysis is based.

S3.2 Model Simulations: Interventions and Tradeoffs.

Complementary dimensionless simulations (Eqs. (S11)–(S15)) were performed to provide generalized insights into the system's structural response to interventions and dynamic changes, independent of absolute scaling. These studies focus on the impact of Non-Invasive Ventilation (NIV) related parameters. Fixed dimensionless parameters for baseline conditions are noted in the figures unless specified otherwise.

We first analyzed the system's response to a temporary enhancement in the NIV recovery rate ($\pi_\theta(\tilde{t}) = \pi_{\theta 0} + \pi_{\Delta\theta}e^{-\pi_\lambda \tilde{t}}$) under constant admission ($\pi_A = 1$), mimicking interventions like short-term staffing surges (Figure S2). The results quantitatively demonstrate the direct clinical benefits of improved NIV efficiency. Increasing the intervention magnitude ($\pi_{\Delta\theta}$) significantly lowers peak and transient normalized NIV occupancy ($\tilde{Y}$, Panel B), thereby reducing the concurrent demand for this resource-intensive therapy. This upstream effect directly translates to improved patient outcomes: cumulative ICU transfers ($\tilde{Z}$, Panel C) and exitus ($\tilde{W}$, Panel D) are substantially mitigated, while cumulative recovery ($\tilde{R}$, Panel E) is accelerated. The phase-space trajectories (Panel F) visually confirm that stronger interventions guide the system towards more favorable outcome states (lower $\tilde{Z}, \tilde{W}$; higher $\tilde{R}$). This underscores the clinical value proposition of the IRCU: targeted improvements in NIV care can dynamically reshape patient flow to optimize resource utilization and patient outcomes during the intervention period.

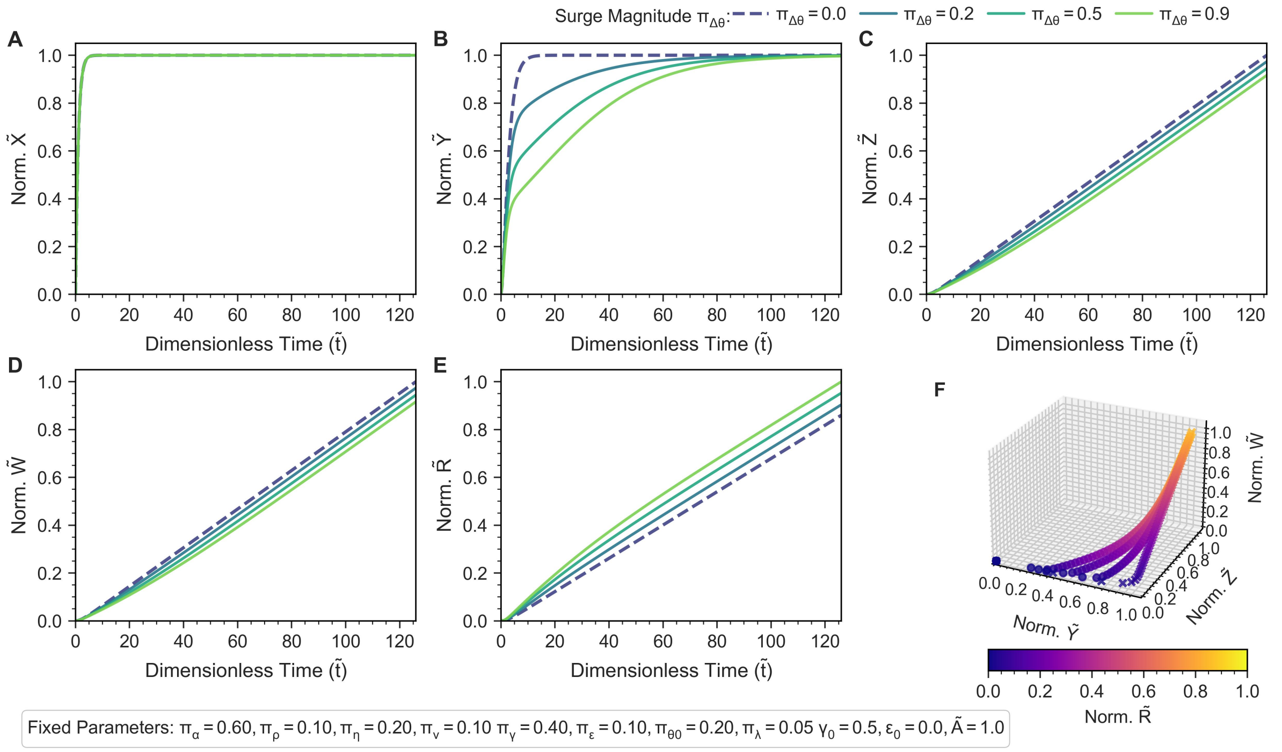

Figure S2| Impact of Transient NIV Recovery Enhancement ($\pi_{\Delta\theta}$) on Dimensionless IRCU Dynamics. (**A-E**) Normalized state variables ($\tilde{X}$-$\tilde{R}$) vs. time ($\tilde{t}$) comparing baseline ($\pi_{\Delta\theta} = 0$, dashed) with scenarios of increasing transient recovery boost ($\pi_{\Delta\theta} > 0$, solid lines). (**F**) Normalized phase-space trajectories ($\tilde{Y}$-$\tilde{Z}$-$\tilde{W}$) colored by normalized recovery ($\tilde{R}$). Enhanced recovery reduces NIV load (B), mitigates adverse outcomes (C, D), and accelerates recovery (E). Fixed simulation parameters noted.

To understand performance under external pressure, we simulated the impact of varying baseline NIV effectiveness during a significant admission surge ($\pi_A(\tilde{t})$ as a Gaussian peak; Figure S3). Three effectiveness levels (Less Effective - LE, Baseline - BE, Highly Effective - HE), defined by concurrently varying $\pi_{\theta 0}, \pi_\gamma, \pi_\epsilon$ (parameters in figure caption), were compared. While the admission surge dominates the temporal pattern of active populations ($\tilde{X}, \tilde{Y}$, Panels B, C), the simulation clearly shows that higher NIV effectiveness (HE) acts as a crucial buffer. It markedly attenuates the negative consequences of the surge on cumulative adverse outcomes, leading to substantially lower ICU transfers ($\tilde{Z}$, Panel D) and exitus ($\tilde{W}$, Panel E), while maximizing recoveries ($\tilde{R}$, Panel F) compared to the LE and BE scenarios [18, 19]. Such effectiveness is recognized to be multifactorial in clinical practice, depending on staff proficiency, protocols, patient selection, and timeliness [20, 21]. This simulation thus highlights that an IRCU's value lies not merely in capacity [22], but significantly in its capability for high-quality care delivery which actively improves outcomes and alleviates downstream ICU pressure during crises [23].

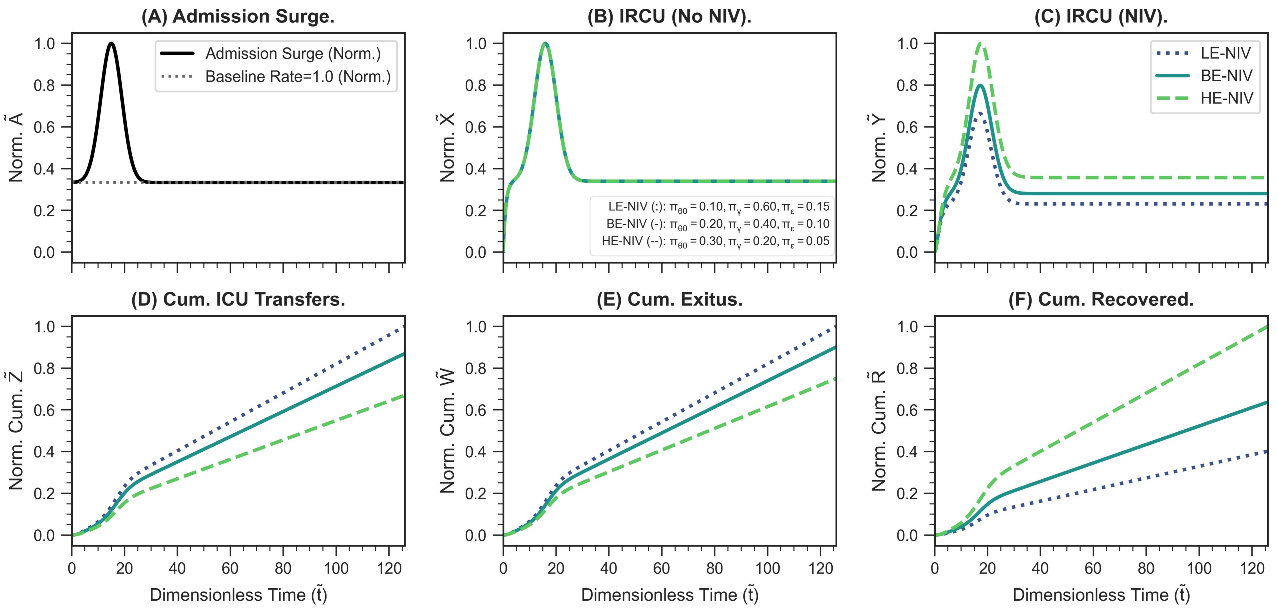

Figure S3| Influence of NIV Effectiveness Level during an Admission Surge. (**A**) Normalized Gaussian admission surge $\tilde{A}(t)$. (**B-F**) Resulting normalized state dynamics ($\tilde{X}$-$\tilde{R}$) for Less Effective (LE, dotted), Baseline (BE, solid), and Highly Effective (HE, dashed) NIV parameter sets (definitions in Panel B legend). Higher effectiveness substantially buffers the surge's impact, reducing adverse outcomes (D, E) while maximizing recovery (F).

We also assessed system resilience to internal fluctuations by simulating a time-varying *NIV recovery rate* ($\pi_\theta(t)$, assuming this is Panel A's variable based on parameters) under constant admission ($\pi_A = 1$) (Figure S4). Comparing LE, BE, and HE effectiveness levels reveals that higher effectiveness not only yields consistently better outcomes (lower $\tilde{W}, \tilde{Z}$; higher $\tilde{R}$) but also enhances system stability. The HE scenario shows dampened oscillations in NIV occupancy ($\tilde{Y}$, Panel C) and mitigated outcome disruptions following the transient change in $\pi_\theta(t)$. This suggests that high-quality NIV care, influenced by clinical expertise, technology, and management strategies [24, 25, 26], is critical not only for managing external load but also for maintaining robust performance despite internal system variability or policy shifts (e.g., evolving ICU admission criteria).

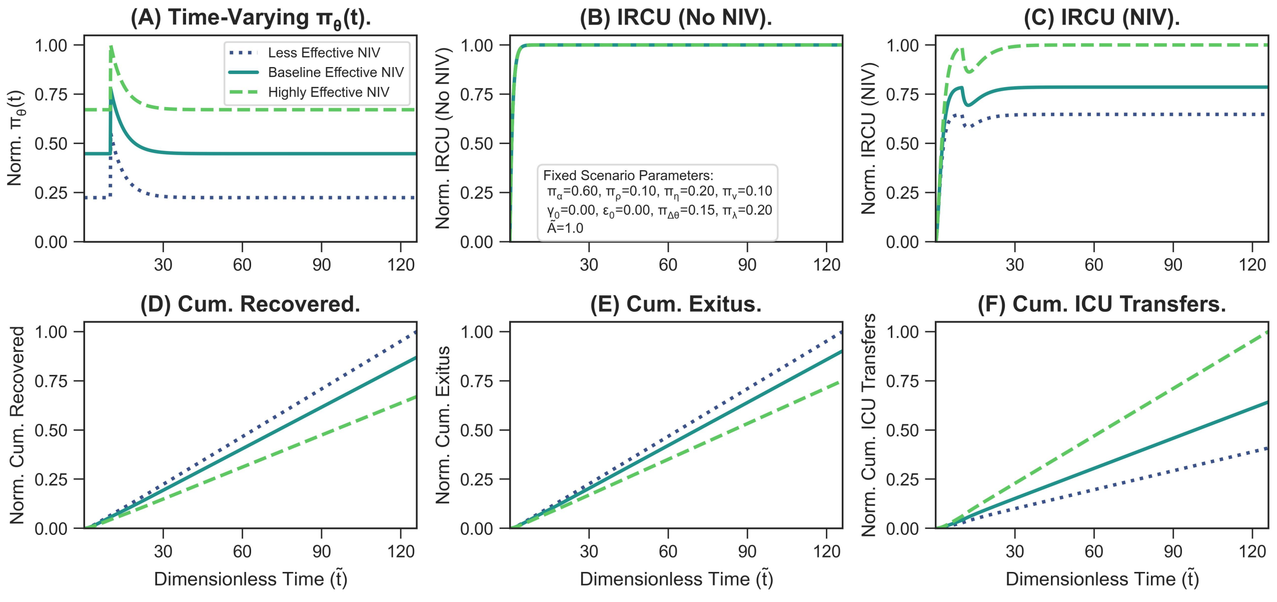

Figure S4| System Response to a Time-Varying NIV Recovery Rate ($\pi_\theta(t)$) across Effectiveness Levels. (**A**) Profile of the simulated transient change in normalized parameter $\pi_\theta(t)$ under constant admission $\tilde{A} = 1$. (**B-F**) Comparison of resulting normalized state dynamics ($\tilde{X}$-$\tilde{Z}$) for Less Effective (LE, dotted), Baseline (BE, solid), and Highly Effective (HE, dashed) NIV effectiveness levels. Higher effectiveness improves overall outcomes and enhances system stability against internal perturbations.

Finally, recognizing that interventions during surges often involve resource tradeoffs, we simulated

the combined effect of simultaneously varying ICU transfer mitigation (γ_0, 0 to 0.5) and NIV mortality reduction (ε_0, 0 to 0.5) effectiveness under admission surge conditions (Figure S5). Plotting the resulting cumulative ICU transfers averted versus cumulative exitus averted reveals the achievable efficiency landscape. The distribution forms a Pareto-like frontier [27], visually representing the tradeoff: prioritizing γ_0 (larger points) maximally averts ICU transfers, while prioritizing ε_0 (brighter colors) maximally averts exitus events. This quantitative tradeoff analysis provides a valuable tool for supporting evidence-informed strategic decision-making [28]. Depending on the primary constraint or objective during a crisis (e.g., preserving ICU capacity vs. minimizing mortality), IRCU leadership can use such insights to target quality improvement efforts or allocate resources more effectively towards protocols impacting either γ_0 or ε_0 [29].

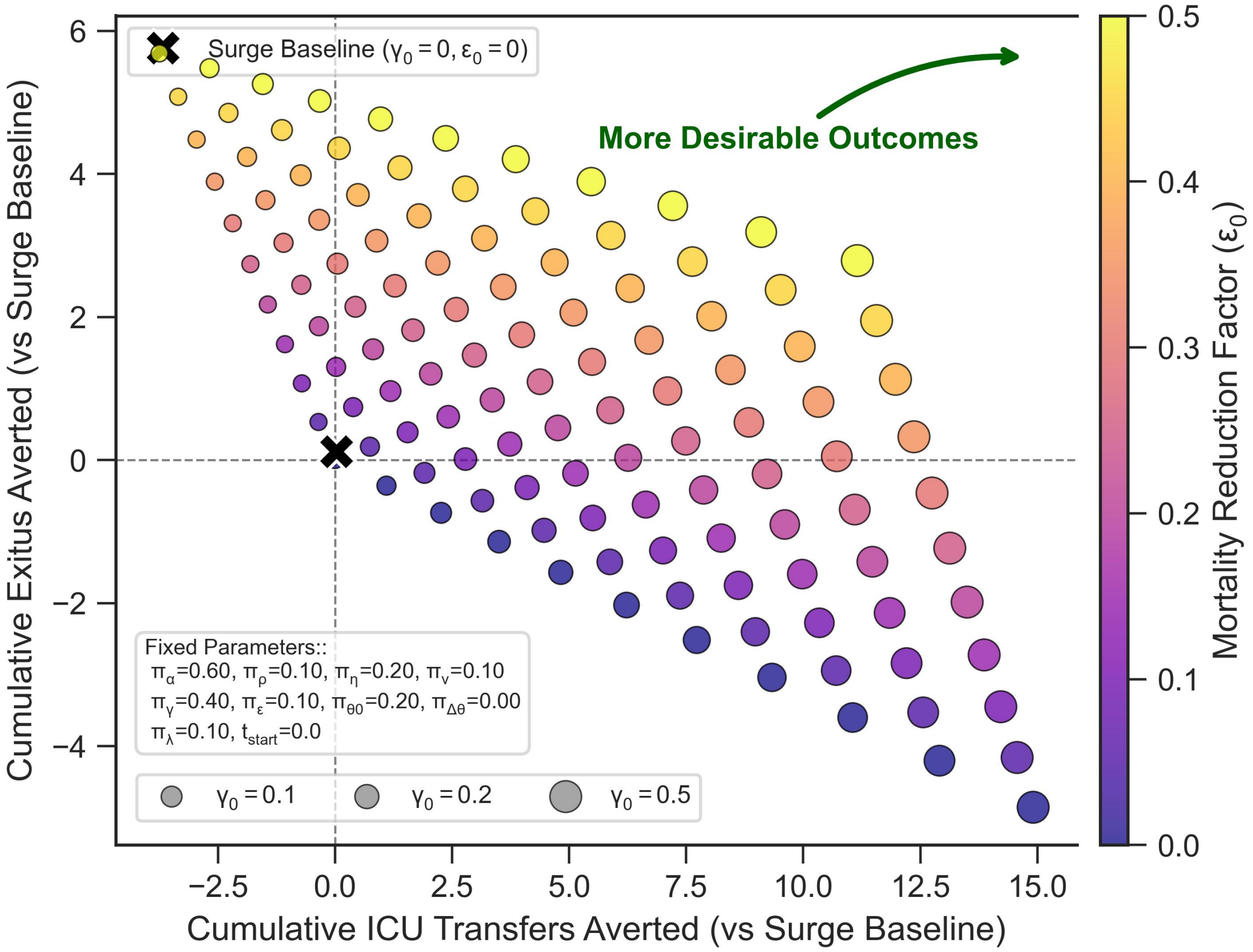

Figure S5| Tradeoffs Between Averting ICU Transfers and Exitus via Combined Interventions During a Surge. Efficiency landscape plotting cumulative ICU transfers averted versus cumulative exitus averted (relative to baseline surge 'X') when simultaneously varying ICU mitigation effectiveness (γ_0, point size, 0-0.5) and mortality reduction effectiveness (ε_0, color, 0-0.5). The frontier illustrates achievable outcomes and the inherent tradeoffs for resource prioritization.

S4 Detailed Description of the LOS/Convolution Occupancy Model.

Accurate forecasting of patient occupancy is essential for effective resource management within Intermediate Respiratory Care Units (IRCUs), particularly given the inherent variability in patient demand and discharge patterns commonly observed in healthcare settings [30, 31]. While traditional queuing models offer analytical tractability, their reliance on strong parametric assumptions (e.g., exponential LOS, Poisson arrivals) often limits their ability to capture the complexities of real-world systems, such as non-standard LOS distributions or time-varying admission rates [31, 32, 33, 34, 35]. To overcome these limitations, we developed a data-driven, probabilistic modeling framework for IRCU occupancy grounded in empirical observations. This approach integrates a non-parametric representation of the Length-of-Stay (LOS) distribution with flexible, stochastic modeling of the admission process using Gaussian Processes (GPs), allowing for robust occupancy prediction and uncertainty quantification.

S4.1 Occupancy Calculation from Empirical LOS and Admissions

The model fundamentally links daily occupancy to past admissions and the duration patients stay. Let d be the LOS in days. Rather than assuming a specific parametric form, we directly characterize the LOS distribution using the empirical Probability Mass Function (PMF) derived from observed patient data:

$$f(d) \;=\; \frac{\text{Number of patients with observed LOS exactly } d}{\text{Total number of observed patients}}, \tag{S16}$$

calculated for all observed LOS durations d up to a maximum $d_{\max}$. This non-parametric method preserves the potentially complex shape (e.g., multimodality, heavy tails) of the observed LOS distribution [7, 36].

From the PMF, the corresponding empirical survival function $S(d)$, representing the probability that a patient admitted on day s is still present in the unit d days later (i.e., $P(\text{LOS} \geq d)$), is calculated as:

$$S(d) \;=\; 1 - \sum_{k=0}^{d-1} f(k). \tag{S17}$$

Note that $S(0) = 1$ by definition.

Let $A(t)$ be the number of new admissions to the IRCU on day t. The total IRCU occupancy $X(t)$ on day t represents the accumulation of patients admitted on or before day t who have not yet been discharged. This relationship is formalized through the discrete convolution of the admission history with the survival function:

$$X(t) \;=\; \sum_{s=0}^{t} A(s)\, S(t-s). \tag{S18}$$

Under steady-state conditions where the admission rate $A(t)$ is constant ($A(t) = A$), this convolution leads to the expected equilibrium occupancy $X = A \times E[\text{LOS}]$, where $E[\text{LOS}]$ is the mean LOS calculated from $f(d)$, linking the model to fundamental queuing principles [16, 9].

Alternatively, the occupancy dynamics can be expressed recursively via a daily flow balance:

$$X(t+1) = X(t) + A(t+1) - L(t+1), \tag{S19}$$

$$L(t+1) = \sum_{d=1}^{d_{\max}} f(d)\, A(t+1-d)\, \mathbb{1}\{t+1-d \geq 0\}, \tag{S20}$$

where $A(t+1)$ are the new admissions during day $t+1$, $L(t+1)$ are the discharges during day $t+1$ (calculated as the sum of patients admitted d days prior who are discharged on day $t+1$, determined by the PMF $f(d)$), and $X(t)$ is the occupancy at the start of day t. The indicator function $\mathbb{1}\{\cdot\}$ ensures causality.

S4.2 Stochastic Admissions Modeling using Gaussian Processes.

Recognizing that daily hospital admissions $A(t)$ are often complex and time-varying, we model them explicitly as draws from a Gaussian Process (GP), a flexible, non-parametric statistical tool suitable for time series [37]. Formally, we represent the admission on day t as:

$$A(t) \;\sim\; \mathcal{GP}(m(t), k(t, t')), \tag{S21}$$

where $m(t)$ is the mean function (often set to zero or a simple trend a priori) and $k(t, t')$ is the covariance or kernel function, which defines the structure and smoothness of the admission patterns. The GP framework inherently treats the underlying admission function as a stochastic process, providing not only a predictive mean $\mu_A(t)$ after conditioning on observed data, but also principled uncertainty quantification via a predictive variance $\sigma_A^2(t)$.

The kernel function $k(t, t')$ specifies the correlation between admissions at different times t and t'. A common initial choice for capturing smooth trends is the Radial Basis Function (RBF) kernel combined with a White Noise kernel to account for independent observation uncertainty:

$$k(t, t') = C \cdot \exp\Big(-\frac{(t-t')^2}{2\ell^2}\Big) + \sigma_n^2\, \delta_{t,t'}, \tag{S22}$$

where C is the amplitude variance, ℓ is the characteristic length-scale governing how quickly correlations decay with time separation, and σ_n^2 is the noise variance. These hyperparameters (collectively $\theta = \{C, \ell, \sigma_n^2\}$) are typically learned from observed admissions data $\mathcal{D} = \{(t_i, A_i)\}$ by maximizing the marginal likelihood.

To better capture specific temporal patterns often present in healthcare admissions, such as weekly seasonality, composite kernels can be constructed by summing basic kernels [38]. For example, incorporating a periodic kernel allows explicit modeling of recurring weekly patterns ($T = 7$ days):

$$k_{\text{comp}}(t, t') = C_1 \cdot \text{RBF}(t, t') + C_2 \cdot \text{Periodic}(t, t'; T = 7) + \sigma_n^2 \delta_{t,t'}, \tag{S23}$$

where C_1, C_2, and parameters within the Periodic kernel (like its own length-scale) are also learned from data. Further enhancements for capturing complex dependencies can involve alternative kernel choices or non-zero structured mean functions $m(t)$, potentially informed by traditional time-series models [38].

By fitting the chosen GP model (i.e., selecting a kernel and optimizing hyperparameters) to historical admission data, we obtain a posterior distribution over the admission function $A(t)$. Sampling trajectories from this posterior and simulating the occupancy dynamics using Eq. (S18) or (S19) for each sample allows the propagation of admission uncertainty through to the occupancy predictions, yielding probabilistic forecasts $X(t)$ with associated credible intervals. This integrated approach leverages the empirical LOS distribution and flexible GP admission modeling to generate occupancy forecasts reflecting system nonlinearities and uncertainties, crucial for robust resource planning [39]. Furthermore, the framework allows for potential integration with real-time data streams and adaptive methods (e.g., Bayesian updating, AI techniques) to dynamically refine $f(d)$ or GP predictions as new information becomes available [12], enhancing its practical utility in dynamic healthcare environments.

S5 ODE Model Parameter Estimation.

The baseline parameters for the ODE model (Eqs. (S6)-(S10)), representing average transition rates [days^{-1}], were calibrated to reflect the observed dynamics of the UHVN IRCU cohort (n=249).

Methodology: The estimation combined observed outcome proportions with assumed average lengths of stay (τ) in the origin states, using the relationship: **Rate $\approx$ Proportion / τ**.

1. **Structural Constraints:** Based on clinical observations (Main Paper Fig. 2B), transitions from the non-NIV state (X) directly to ICU (Z) or Exitus (W) were non-existent in this cohort. Thus, $\eta = 0$ and $\nu = 0$ were fixed. Intervention modulators γ_0, ε_0 were set to 0 for baseline estimation.

2. **Outcome Proportions Calculation:** Proportions were calculated directly from the patient cohort data:

   - From State X (N=249): $P_{X\to Y} = 77/249 \approx 0.3092$ (Initiated NIV), $P_{X\to R} = 172/249 \approx 0.6908$ (Recovered without NIV).
   - From State Y (n=77 initiated NIV): $P_{Y\to Z} = 18/77 \approx 0.2338$ (Transferred to ICU), $P_{Y\to W} = 7/77 \approx 0.0909$ (Exitus, no prior ICU transfer), $P_{Y\to R} = 52/77 \approx 0.6753$ (Recovered from NIV).

3. **Assumed LOS for Rate Calculation:** Lacking granular time-in-state data, we used assumed average LOS values as the characteristic time τ for transitions, consistent with parameters used in the simulation analyses: $\tau_X = 7$ days (Avg. time in State X before transition), $\tau_Y = 10$ days (Avg. time in State Y before transition). This is a necessary simplifying assumption for this modeling approach.

4. **Rate Calculation:** $\alpha \approx P_{X\to Y}/\tau_X \approx 0.3092/7 \approx 0.04418$ days^{-1}, $\rho \approx P_{X\to R}/\tau_X \approx 0.6908/7 \approx 0.09868$ days^{-1}, $\gamma \approx P_{Y\to Z}/\tau_Y \approx 0.2338/10 = 0.02338$ days^{-1}, $\varepsilon \approx P_{Y\to W}/\tau_Y \approx 0.0909/10 \approx 0.00909$ days^{-1}, $\theta_0 \approx P_{Y\to R}/\tau_Y \approx 0.6753/10 \approx 0.06753$ days^{-1}.

The resulting baseline parameters, consistent with total outflow rates $(\alpha + \rho) \approx 1/\tau_X$ and $(\gamma + \varepsilon + \theta_0) \approx 1/\tau_Y$, are summarized in Table S1.

Table S1| Baseline ODE model parameters estimated from cohort data and analysis assumptions.

Parameter	Description	Estimated Value [days^{-1}]
α	Rate X → Y (NIV Initiation)	0.04418
ρ	Rate X → R (Direct Recovery)	0.09868
η	Rate X → Z (Direct ICU)	0 (Fixed by observation)
ν	Rate X → W (Direct Exitus)	0 (Fixed by observation)
γ	Rate Y → Z (ICU from NIV)	0.02338
ε	Rate Y → W (Exitus from NIV)	0.00909
θ_0	Rate Y → R (Recovery from NIV)	0.06753
Parameters assumed for estimation:		
τ_X	Assumed Avg. LOS in State X	7 days
τ_Y	Assumed Avg. LOS in State Y	10 days

Note: These are baseline estimates for the autonomous model. For simulations involving time-varying recovery (e.g., Main Paper Fig. 3G-I, J-L), the rate $\theta(t)$ was modified as described. Intervention modulators γ_0, ε_0 were set to 0 for baseline.

S6 Appendix: Model Variables and Parameters.

Table S2| Summary of the model's dynamic state variables.

Variable	Description [Unit]
$X(t)$	Patients in IRCU, not receiving NIV at time t [patients]
$Y(t)$	Patients in IRCU, actively receiving NIV at time t [patients]
$Z(t)$	Patients transferred from IRCU pathway to ICU by time t [patients]
$W(t)$	Exitus from IRCU pathway (X or Y) by time t [patients]
$R(t)$	Recovered (discharged/to ward) from IRCU pathway (X or Y) by time t [patients]

Table S3| Summary of the model parameters including intervention modulators.

Parameter	Description [Unit]
Admission	
$A(t)$	Influx rate of new patient admissions to IRCU [patients/time]
Transitions from Non-NIV State (X)	
α	Rate of NIV initiation (X → Y) [time^{-1}]
ρ	Direct recovery/discharge rate (from X) [time^{-1}]
η	Direct ICU transfer rate (from X) [time^{-1}]
ν	Direct exitus rate (from X) [time^{-1}]
Baseline Transitions from NIV State (Y)	
γ	Baseline rate of ICU transfer (from Y) [time^{-1}]
ε	Baseline rate of exitus (from Y) [time^{-1}]
θ_0	Baseline rate of recovery (from Y) [time^{-1}]
Intervention Modulators (acting on Y transitions)	
γ_0	ICU transfer mitigation factor (effectiveness) [dimensionless]
ε_0	Exitus reduction factor (effectiveness) [dimensionless]
$\Delta\theta$	Initial magnitude of transient recovery enhancement [time^{-1}]
λ	Decay rate of transient recovery enhancement [time^{-1}]

References

[1] A. Esteban, F. Frutos-Vivar, A. Muriel, et al. "Evolution of Mortality over Time in Patients Receiving Mechanical Ventilation". In: *American Journal of Respiratory and Critical Care Medicine* 188.2 (2013), pp. 220–230. DOI: 10.1164/rccm.201212-2169OC.

[2] L. Cabrini, G. Landoni, A. Oriani, et al. “Noninvasive ventilation and survival in acute care settings: a comprehensive systematic review and metaanalysis of randomized controlled trials”. In: *Critical Care Medicine* 43.4 (2015), pp. 880–888. DOI: 10.1097/CCM.0000000000000819.

[3] V. Lemiale, D. Mokart, M. Resche-Rigon, et al. “Effect of Noninvasive Ventilation vs Oxygen Therapy on Mortality Among Immunocompromised Patients With Acute Respiratory Failure: A Randomized Clinical Trial”. In: *JAMA* 314.16 (2015), pp. 1711–1719. DOI: 10.1001/jama.2015.12402.

[4] G. Hernández, C. Vaquero, L. Colinas, et al. “Effect of Postextubation High-Flow Nasal Cannula vs Conventional Oxygen Therapy on Reintubation in Low-Risk Patients: A Randomized Clinical Trial”. In: *JAMA* 315.13 (2016), pp. 1354–1361. DOI: 10.1001/jama.2016.2711.

[5] L. H. Aiken, D. M. Sloane, L. Bruyneel, et al. “Nurse staffing and education and hospital mortality in nine European countries: a retrospective observational study”. In: *The Lancet* 383.9931 (2014), pp. 1824–1830. DOI: 10.1016/S0140-6736(13)62631-8.

[6] P. Griffiths, J. Ball, K. Bloor, et al. “Nurse staffing levels, missed vital signs and mortality in hospitals: retrospective longitudinal observational study”. In: (2018). DOI: 10.3310/hsdr06380.

[7] J. D. Murray. *Mathematical Biology I: An Introduction*. 3rd. Springer, 2002.

[8] M. J. Keeling and P. Rohani. *Modeling Infectious Diseases in Humans and Animals*. Princeton University Press, 2008. DOI: 10.1515/9781400841035.

[9] O. Diekmann, H. Heesterbeek, and T. Britton. *Mathematical Tools for Understanding Infectious Disease Dynamics*. Princeton University Press, 2013.

[10] T. Al-Karkhi and K. Byatt. “A compartmental model to describe acute medical in-patient flow through a hospital”. In: *Heliyon* 11.3 (2025), e42260. DOI: 10.1016/j.heliyon.2025.e42260.

[11] J. Garrido, D. Martínez-Rodríguez, F. Rodríguez-Serrano, et al. “Mathematical model optimized for prediction and health care planning for COVID-19”. In: *Medicina Intensiva (English Edition)* 46.5 (2022), pp. 248–258. DOI: https://doi.org/10.1016/j.medine.2022.02.020.

[12] H. T. Banks and K. Kunisch. *Estimation Techniques for Distributed Parameter Systems*. Birkhäuser, 1989. DOI: 10.1007/978-1-4612-3700-6.

[13] O. Diekmann and J. A. P. Heesterbeek. *Mathematical Epidemiology of Infectious Diseases: Model Building, Analysis and Interpretation*. John Wiley & Sons, 2000.

[14] F. Brauer. “Mathematical epidemiology: Past, present, and future”. In: *Infectious Disease Modelling* 2.2 (2017), pp. 113–127. DOI: 10.1016/j.idm.2017.02.001.

[15] L. J. S. Allen. *An Introduction to Stochastic Processes with Applications to Biology*. Pearson Education, 2003.

[16] S. M. Ross. *Introduction to Probability Models*. 11th. Academic Press, 2014.

[17] N. Bacaër. *A Short History of Mathematical Population Dynamics*. Springer, 2011. DOI: 10.1007/978-0-85729-115-8.

[18] B. Rochwerg, L. Brochard, M. W. Elliott, et al. “Official ERS/ATS clinical practice guidelines: noninvasive ventilation for acute respiratory failure”. In: *European Respiratory Journal* 50.2 (2017), p. 1602426. DOI: 10.1183/13993003.02426-2016.

[19] R. Scala and L. Pisani. “Non-invasive ventilation in acute respiratory failure: Which recipe?” In: *European Respiratory Review* 27.150 (2018), p. 180029. DOI: 10.1183/16000617.0029-2018.

[20] E. Ozyilmaz, A. O. Ugurlu, and S. Nava. “Timing of noninvasive ventilation failure: causes, risk factors, and potential remedies”. In: *BMC Pulmonary Medicine* 14.1 (2014), p. 19. DOI: 10.1186/1471-2466-14-19.

[21] I. Ruzsics, P. Matrai, P. Hegyi, et al. “Noninvasive ventilation improves the outcome in patients with pneumonia-associated respiratory failure: Systematic review and meta-analysis”. In: *Journal of Infection and Public Health* 15.3 (2022), pp. 349–359. DOI: 10.1016/j.jiph.2022.02.004.

[22] M. Prin and H. Wunsch. “The role of stepdown beds in hospital care”. In: *American Journal of Respiratory and Critical Care Medicine* 190.11 (2014), pp. 1210–1216. DOI: 10.1164/rccm.201406-1117PP.

[23] S. A. Nasraway, I. L. Cohen, R. C. Dennis, et al. “Guidelines on admission and discharge for adult intermediate care units”. In: *Critical Care Medicine* 26.3 (1998), pp. 607–610. DOI: 10.1097/00003246-199803000-00039.

[24] A. M. Esquinas, ed. *Noninvasive Mechanical Ventilation. Theory, Equipment, and Clinical Applications.* Publicado el 1 de enero de 2010. Springer Berlin Heidelberg, 2010, p. 402. DOI: 10.1007/978-3-642-11365-9.

[25] A. M. Esquinas, ed. *Noninvasive Mechanical Ventilation. Theory, Equipment, and Clinical Applications.* 2nd ed. Springer Cham, 2016, p. 959. DOI: 10.1007/978-3-319-21653-9.

[26] A. M. Esquinas, ed. *Noninvasive Mechanical Ventilation. Theory, Equipment, and Clinical Applications.* 3rd ed. Publicado el 28 de agosto de 2023. Springer Cham, 2023, pp. XXXVII, 847. DOI: 10.1007/978-3-031-28963-7.

[27] K. Deb. “Multi-Objective Optimization”. In: *Search Methodologies: Introductory Tutorials in Optimization and Decision Support Techniques.* Ed. by E. K. Burke and G. Kendall. Boston, MA: Springer US, 2005, pp. 273–316. DOI: 10.1007/0-387-28356-0_10.

[28] M. L. Brandeau, F. Sainfort, and W. P. Pierskalla, eds. *Operations Research and Health Care. A Handbook of Methods and Applications.* 1st ed. International Series in Operations Research & Management Science. New York, NY: Springer New York, 2004, pp. VIII, 874. DOI: 10.1007/b106574.

[29] C. Kahraman and Y. I. Topcu, eds. *Operations Research Applications in Health Care Management.* 1st ed. International Series in Operations Research & Management Science 255. Cham: Springer Cham, 2018, pp. XV, 604. DOI: 10.1007/978-3-319-65455-3.

[30] E. Litvak and M. Bisognano. “More patients, less payment: increasing hospital efficiency in the aftermath of health reform”. In: *Health Affairs (Millwood)* 30.1 (2011), pp. 76–80. DOI: 10.1377/hlthaff.2010.1114.

[31] R. Hall, ed. *Patient Flow: Reducing Delay in Healthcare Delivery.* 2nd ed. International Series in Operations Research & Management Science. Springer New York, NY, 2013, pp. XII, 553. DOI: 10.1007/978-1-4614-9512-3.

[32] J. B. Jun, S. H. Jacobson, and J. R. Swisher. “Application of Discrete-Event Simulation in Health Care Clinics: A Survey”. In: *The Journal of the Operational Research Society* 50.2 (1999), pp. 109–123. DOI: 10.2307/3010560.

[33] D. Fone, S. Hollinghurst, M. Temple, et al. “Systematic review of the use and value of computer simulation modelling in population health and health care delivery”. In: *Journal of Public Health* 25.4 (2003), pp. 325–335. DOI: 10.1093/pubmed/fdg075.

[34] S. H. Jacobson, S. N. Hall, and J. R. Swisher. “Discrete-Event Simulation of Health Care Systems”. In: *Patient Flow: Reducing Delay in Healthcare Delivery.* Ed. by R. W. Hall. Boston, MA: Springer US, 2006, pp. 211–252. DOI: 10.1007/978-0-387-33636-7_8.

[35] J. D. Griffiths, N. Price-Lloyd, M. Smithies, et al. “A queueing model of activities in an intensive care unit”. In: *IMA Journal of Management Mathematics* 17.3 (2006), pp. 277–288. DOI: 10.1093/imaman/dpi042.

[36] J. M. Heffernan, R. J. Smith, and L. M. Wahl. “Perspectives on the basic reproductive ratio”. In: *Journal of the Royal Society Interface* 2.4 (2005), pp. 281–293. DOI: 10.1098/rsif.2005.0042.

[37] C. E. Rasmussen and C. K. I. Williams. *Gaussian Processes for Machine Learning.* The MIT Press, 2005. DOI: 10.7551/mitpress/3206.001.0001.

[38] S. Roberts, M. Osborne, M. Ebden, et al. “Gaussian processes for time-series modelling”. In: *Philosophical Transactions of the Royal Society A: Mathematical, Physical and Engineering Sciences* 371.1984 (2013), p. 20110550. DOI: 10.1098/rsta.2011.0550.

[39] W. Whitt and X. Zhang. “Forecasting arrivals and occupancy levels in an emergency department”. In: *Operations Research for Health Care* 21 (2019), pp. 1–18. DOI: 10.1016/j.orhc.2019.01.002.
